# Global burden of hepatitis C virus infection related to high body mass index and future forecast: an analysis based on the global burden of disease study 2021

**DOI:** 10.3389/fpubh.2025.1685807

**Published:** 2025-11-20

**Authors:** Jiayi Chen, Shiyun Wu, Panpan Zhai, Xueting Ou, Liyang Zhou, Xingfei Pan

**Affiliations:** Guangdong Provincial Key Laboratory of Major Obstetric Diseases, Department of Infectious Diseases, Guangdong Provincial Clinical Research Center for Obstetrics and Gynecology, The Third Affiliated Hospital, Guangzhou Medical University, Guangzhou, China

**Keywords:** hepatitis C virus, body mass index, global burden of disease, socio-demographic index, World Health Organization

## Abstract

**Objective:**

The global prevalence of obesity is rising, and prior research has established a strong link between obesity and hepatitis C prognosis. However, the impact of high body mass index (HBMI) on the HCV burden remains uncertain. This study sought to clarify the overall HCV burden related to HBMI and examine temporal trends.

**Methods:**

Public data from the Global Burden of Disease (GBD) database (1990–2021) were utilized to analyze the global and different Socio-demographic index (SDI) regional burden of HCV associated with obesity, focusing on Deaths, Disability-Adjusted Life Years (DALYs), Years Lived with Disability (YLDs), and Years of Life Lost (YLLs). Trends in the HCV burden were assessed using Estimated Annual Percentage Changes (EAPCs) and Average Annual Percentage Changes (AAPCs) via Joinpoint regression. The age-period-cohort (APC) model was used to examine the effects of age, period, and cohort on disease burden, respectively. The Das Gupta decomposition analysis method was applied to evaluate the contributions of population growth, population aging, and epidemiological changes to the burden. Frontier analysis was conducted to explore the relationship between the HCV burden linked to HBMI and Socio-demographic development. An ARIMA model was then developed to forecast the Age-standardized Mortality Rate (ASMR) and Age-standardized DALYs Rate (ASDR) of HBMI-associated hepatitis C over the next 15 years.

**Results:**

From 1990 to 2021, global HCV deaths related to obesity rose from 3,835 to 17,090, with DALYs increasing from 94,503 to 389,263. The EAPCs for ASMR and ASDR were 2.20 and 2.10, respectively. Obesity posed a greater burden on female patients infected with HCV virus compared to males. In terms of age, the effect of HBMI on HCV patients increased with age. Over the past 30 years, ASMR and ASDR have consistently risen across all SDI regions (All regions: EAPCs > 0, 95% CIs > 0). The High SDI region reported the highest deaths, DALYs, ASMR, and ASDR annually, indicating the greatest obesity impact on HCV burden of this area. However, obesity also had an increasingly large impact on the HCV disease burden in the Middle SDI and Low-middle SDI regions. Regionally, Africa, the Middle East, and Central Asia bear a relatively heavy burden of HCV associated with HBMI, and the burden in North America and Oceania cannot be ignored. At the national level, Mongolia and Egypt have the heaviest burden. The results of decomposition analysis show that epidemiological changes are the main cause of the increased burden. Projections suggest a continued increase in the obesity-related HCV burden globally and across different SDI regions over the next 15 years.

**Conclusion:**

Obesity poses an increasing disease burden for people infected with hepatitis C virus. Targeted public health interventions are urgently needed to alleviate this burden.

## 1 Introduction

Hepatitis C remains a critical global health challenge and a leading risk factor for liver cirrhosis and cancer (1). The advent of pan-genotypic direct-acting antiviral agents (DAAs) has reduced the global incidence of hepatitis C virus (HCV) infections, with approximately 250,000 fewer cases annually between 2015 and 2019 (2, 3). Nonetheless, only 36% of individuals with chronic HCV have been diagnosed, and a mere 20% have commenced treatment (4). According to the latest WHO data, an estimated 50 million people globally are living with chronic hepatitis C virus infection, with about 1 million new cases emerging annually. In 2022, approximately 242,000 individuals died from hepatitis C, primarily due to liver cirrhosis and hepatocellular carcinoma (HCC).^1^ Consequently, these figures highlight a significant gap in achieving the WHO’s target of eliminating viral hepatitis by 2030 (4, 5).

Cirrhosis and hepatocellular carcinoma are the leading causes of mortality in chronic hepatitis C patients (6). Factors such as age, gender, alcohol consumption, smoking, obesity, diabetes, HCV genotype, and co-infection with HIV or HBV can accelerate the progression of hepatitis C (7–10). Previous studies have shown that obesity exacerbates liver fibrosis in HCV patients and accelerates progression to hepatocellular carcinoma (9–11). The mechanism is mainly related to the fact that the HCV virus can cause lipid metabolism disorders and obesity can lead to visceral fat toxicity in HCV patients (12). Those with both CHC and steatosis face a higher risk of HCC and mortality compared to those without steatosis (13). A French longitudinal cohort study on HCV-related cirrhosis demonstrated a dose–response relationship between BMI and HCC risk: a BMI of 25–30 had a hazard ratio of 1.7 for HCC, while a BMI of 30 kg/m^2^ had a hazard ratio of 2.9 (14). Thus, obesity is strongly linked to the prognosis of HCV-infected patients.

Obesity, driven by shifts in socioeconomic factors, dietary habits, and lifestyles, poses a significant global health challenge. By 2021, an estimated 1 billion adult men and 1.11 billion adult women were overweight or obese (15). This trend has risen universally, with no country reversing it (15). Obesity exacerbates conditions like diabetes and cardiovascular diseases and significantly contributes to liver damage, accelerating liver cirrhosis and hepatocellular carcinoma (10, 16, 17). The GBD report highlights obesity as one of the factors in hepatitis C—related liver cancer deaths, rising from third in 1990 to second in 2021, surpassing smoking and excessive alcohol consumption (Supplement 1 in Supplementary material). Therefore, there is an urgent need for long-term policies to mitigate obesity’s impact on hepatitis C prognosis.

## 2 Materials and methods

### 2.1 Data sources

The GBD database evaluates the impact of 300+ diseases, injuries, and 88 risk factors across 204 countries over the last three decades, stratified by gender, age, and age groups. Detailed methodology is available on the official website.^2^ Our study utilized the 2021 GBD data from the Global Health Data Exchange GBD Results Tool.^3^ The specific search methods encompass: (1) “High body-mass index” as risk factor; (2) “Total burden related to hepatitis C” as cause; (3) Measures including Deaths, DALYs, YLDs, and YLLs; (4) Metrics comprising Number and Rate; (5) Data collected from three sex groups (male, female, and both) and all age groups (including Age-standardized); (6) Data spanning from 1990 to 2021; (7) Regional coverage encompassing global, 204 countries, and 5 distinct SDI regions; (8) Finally, search and download the summary table of SDI values for each country on the GBD data source website.

### 2.2 Definition

The total burden related to hepatitis C is defined as the deaths and disabilities caused by acute HCV infection, as well as the overall burden of liver cirrhosis and liver cancer resulting from chronic HCV infection (18). The SDI value serves as a comprehensive indicator of a nation’s development status, taking into account factors such as fertility rates, educational attainment, and per capita income (19). Its scale ranges from 0 to 1, with a higher value indicating greater socioeconomic advancement (20). Specifically, it can be divided into High SDI (SDI ≥ 0.805129), High-middle SDI (0.689504 ≤ SDI < 0.805129), Middle SDI (0.607679 ≤ SDI < 0.689504), Low-middle SDI (0.454743 ≤ SDI < 0.607679), and Low SDI (SDI < 0.454743) (20).

For each indicator of the disease burden (including Deaths, DALYs, YLDs, YLLs), the data are recorded as numbers and age-standardized rates (per 100,000 population). YLDs stands for years lived with disability. It measures the amount of time people lose to diseases and injuries that degrade health but do not cause death. It is calculated by multiplying a disability’s severity by the time it lasts. YLLs stands for years of life lost. It is a measure of premature death within a group of people. YLLs are calculated by starting with the highest achievable life expectancy in a given year for a given age group, then subtracting the age at which a person in that age group dies. DALYs stands for disability-adjusted life years. It refers to the total “healthy” years lost in a population due to disability or death from a specific cause, serving as a comprehensive measure of overall health loss. DALYs are computed as the sum of YLDs and YLLs. The age-standardized rate (ASR) is an adjusted rate obtained by multiplying the disease’s crude rate at a specific age by the age weight (the proportion of the population in each age group) and summing these weighted rates across all age groups.

### 2.3 Statistical analysis

We statistically characterized the total hepatitis C burden linked to HBMI in 2021, stratified by gender and age. We also plotted the trends in disease burden from 1990 to 2021 globally and across various SDI regions. Additionally, we examined the relationship between the HCV burden associated with HBMI and the SDI index across different countries. Spearman’s correlation coefficient assessed the correlations between ASMR, ASDR, and SDI, respectively.

We employed the EAPC and the AAPC to characterize the average annual change rate of the ASR over a specific time frame. A positive EAPC value along with a 95% CI greater than 0, indicates an increasing trend in the ASR, while a negative EAPC value with a 95% CI below 0, signifies a decreasing trend. If the 95% CI of the EAPC encompasses 0, it suggests a stable trend in the ASR. The interpretation for AAPC values followed the same criteria.

We employed the age-period-cohort (APC) model to assess the impacts of age, period, and cohort on mortality related to HBMI-associated HCV. The longitudinal age curve, reflecting the aging process (age effect), was estimated as the expected age-specific mortality rate in the reference cohort adjusted for period deviations. The period rate ratio (RR), defined as the ratio of mortality rates in each period to the reference one, was adopted as an indicator of the period effect, which referred to the long-term trends caused by changes in environments and occurred in all ages concurrently. The cohort RR, i.e., the ratio of mortality rates in each cohort to the reference one, was employed to evaluate variations in mortality across generations (cohort effect). Besides, Net drift and local drift were calculated to assess annual percentage changes in the expected age-adjusted mortality rate and changes in the expected age-specific mortality rate over time, respectively. The specific methods mentioned above refer to previous studies (21, 22). To construct the age-period-cohort model, we divided the data series into consecutive 5-year intervals from 1992 to 2021. Data from 1990 to 1991 were not analyzed as they did not span a 5-year interval. Age was also grouped into 5-year age groups, ranging from 20–24 years to 95 + years. Individuals aged < 20 years were excluded from the analysis due to the low risk of HCV associated with HBMI. In addition, 16 cohorts were summarized, covering subjects born from 1900 to 2000. The mean level of age, period and cohort was selected as the reference groups.

We applied the Das Gupta decomposition analysis method to comprehensively assess the contributions to the increase in the number of deaths and DALYs associated with HBMI-related HCV from 1990 to 2021. These determinants include the effects of population growth, population aging, and epidemiological changes. Epidemiological changes refer to age - and population - adjusted mortality rates and DALY rates at the baseline level (23).

Frontier analysis was applied to assess the association between the overall burden of HCV linked to HBMI and sociodemographic development. A non-linear frontier was constructed using non-parametric data envelopment analysis, with detailed methodologies drawn from prior research (24). This frontier represents the minimum achievable burden determined by the development status (24). The absolute distance between a country’s observed ASMR or ASDR and its frontier is defined as the effective difference. A larger effective difference signifies a greater potential for improvement in HCV management within the country, indicating that these nations could mitigate the HCV burden associated with obesity by optimizing policies and resource allocation.

Finally, an ARIMA model was developed to forecast the future trends of ASMR and ASDR of HCV related to HBMI over a 15-year period. By integrating autoregression (AR) and moving average (MA) components, this model adeptly captures temporal trends and seasonal fluctuations in data (25). In general, the model is expressed as ARIMA (p, d, q), p means the order of auto-regression, d means the order of difference and q means the order of moving average (25).

Data analysis and visualization in this study utilized R statistical software (version 4.4.3) and Joinpoint software (version 5.4.0). Statistical significance was defined as a *p*-value < 0.05.

## 3 Results

### 3.1 Total burden of hepatitis C associated with HBMI globally and in different SDI regions

Globally, the number of hepatitis C deaths associated with HBMI rose from 3,835 in 1990 to 17,090 in 2021, a 3.46-fold increase. The ASMR exhibited an EAPC of 2.20 (95% CI: 2.09, 2.31). DALYs reached 389,263 in 2021, a 3.12-fold rise from 94,503 in 1990. ASDR has an EAPC of 2.10 (95% CI: 1.98, 2.22). Regionally, obesity-related hepatitis C Deaths and DALYs increased significantly in all SDI regions in 2021 compared with 1990. The EAPC values for ASMR/ASDR, and their 95% CIs all exceeded 0, indicating a significant upward trend. In both 1990 and 2021, the High SDI region reported the highest numbers of deaths (1990: 1,413; 2021: 6,109) and DALYs (1990: 32,987; 2021: 124,257) among all regions. However, the Middle SDI region exhibited the most rapid growth rates for ASMR and ASDR globally. Specifically, the EAPC values for ASMR and ASDR were 2.89 (95% CI: 2.81, 2.98) and 2.81 (95% CI: 2.73, 2.88), respectively, representing the highest values observed across all regions. [Table 1].

**Table 1:** Total burden of hepatitis C associated with HBMI globally and in different SDI regions.

Location	Measure	1990		2021		1990–2021 EAPC
		All-ages cases	Age-standardized rates per 100,000 people	All-ages cases	Age-standardized rates per 100,000 people	
		*n* (95% UI)	*n* (95% UI)	*n* (95% UI)	*n* (95% UI)	*n* (95% CI)
Global	Deaths	3,835 (1,537, 6,433)	0.1 (0.04, 0.17)	17,090 (7,185, 28,885)	0.2 (0.08, 0.34)	2.20 (2.09, 2.31)
	DALYs	94,503 (38,104, 160,785)	2.35 (0.94, 3.98)	389,263 (162,464, 665,734)	4.46 (1.86, 7.62)	2.10 (1.98, 2.22)
	YLDs	872 (325, 1,534)	0.02 (0.01, 0.04)	4,192 (1,646, 7,506)	0.05 (0.02, 0.09)	
	YLLs	93,631 (37,752, 159,124)	2.32 (0.93, 3.94)	385,071 (160,949, 658,116)	4.41 (1.84, 7.53)	
Low SDI	Deaths	131 (47, 243)	0.06 (0.02, 0.11)	486 (179, 853)	0.1 (0.04, 0.17)	1.47 (1.38, 1.56)
	DALYs	3,639 (1,342, 6,800)	1.5 (0.55, 2.82)	13,480 (4,974, 23,803)	2.47 (0.92, 4.32)	1.42 (1.34, 1.51)
	YLDs	28 (9, 56)	0.01 (0, 0.02)	104 (37, 194)	0.02 (0.01, 0.04)	
	YLLs	3,611 (1,333, 6,744)	1.49 (0.54, 2.79)	13,375 (4,941, 23,633)	2.45 (0.91, 4.28)	
Low-middle SDI	Deaths	679 (246, 1,291)	0.12 (0.04, 0.22)	3,235 (1,384, 5,477)	0.23 (0.1, 0.38)	2.53 (2.34, 2.71)
	DALYs	18,420 (6,797, 34,901)	2.87 (1.04, 5.44)	86,501 (36,186, 146,860)	5.71 (2.41, 9.68)	2.66 (2.43, 2.88)
	YLDs	140 (50, 271)	0.02 (0.01, 0.04)	673 (269, 1,212)	0.05 (0.02, 0.08)	
	YLLs	18,279 (6,742, 34,687)	2.85 (1.03, 5.41)	85,828 (35,875, 145,635)	5.67 (2.39, 9.6)	
Middle SDI	Deaths	543 (222, 915)	0.06 (0.02, 0.1)	3,547 (1,491, 6,205)	0.14 (0.06, 0.24)	2.89 (2.81, 2.98)
	DALYs	14,159 (5,816, 24,028)	1.33 (0.54, 2.26)	84,403 (34,587, 147,363)	3.06 (1.26, 5.32)	2.81 (2.73, 2.88)
	YLDs	116 (42, 206)	0.01 (0, 0.02)	806 (311, 1,458)	0.03 (0.01, 0.05)	
	YLLs	14,043 (5,774, 23,844)	1.32 (0.54, 2.24)	83,596 (34,272, 145,690)	3.03 (1.25, 5.26)	
High-middle SDI	Deaths	1,064 (440, 1,811)	0.11 (0.05, 0.19)	3,700 (1,484, 6,619)	0.19 (0.07, 0.33)	1.58 (1.48, 1.69)
	DALYs	25,187 (10,310, 43,330)	2.49 (1.02, 4.27)	80,338 (32,047, 143,917)	4.02 (1.61, 7.18)	1.46 (1.35, 1.56)
	YLDs	230 (85, 415)	0.02 (0.01, 0.04)	871 (322, 1,621)	0.04 (0.02, 0.08)	
	YLLs	24,957 (10,221, 42,942)	2.47 (1.01, 4.23)	79,467 (31,715, 142,392)	3.98 (1.59, 7.09)	
High SDI	Deaths	1,413 (587, 2,395)	0.13 (0.05, 0.22)	6,109 (2,574, 10,209)	0.28 (0.12, 0.47)	2.40 (2.14, 2.66)
	DALYs	32,987 (13,649, 55,772)	3.05 (1.27, 5.17)	124,257 (52,296, 210,643)	6.21 (2.6, 10.51)	2.18 (1.92, 2.44)
	YLDs	357 (140, 635)	0.03 (0.01, 0.06)	1,734 (689, 3,068)	0.08 (0.03, 0.15)	
	YLLs	32,631 (13,492, 55,180)	3.02 (1.25, 5.12)	122,523 (51,651, 207,422)	6.13 (2.57, 10.36)	

### 3.2 The distribution map of HCV burden related to HBMI

**Figure 1:**
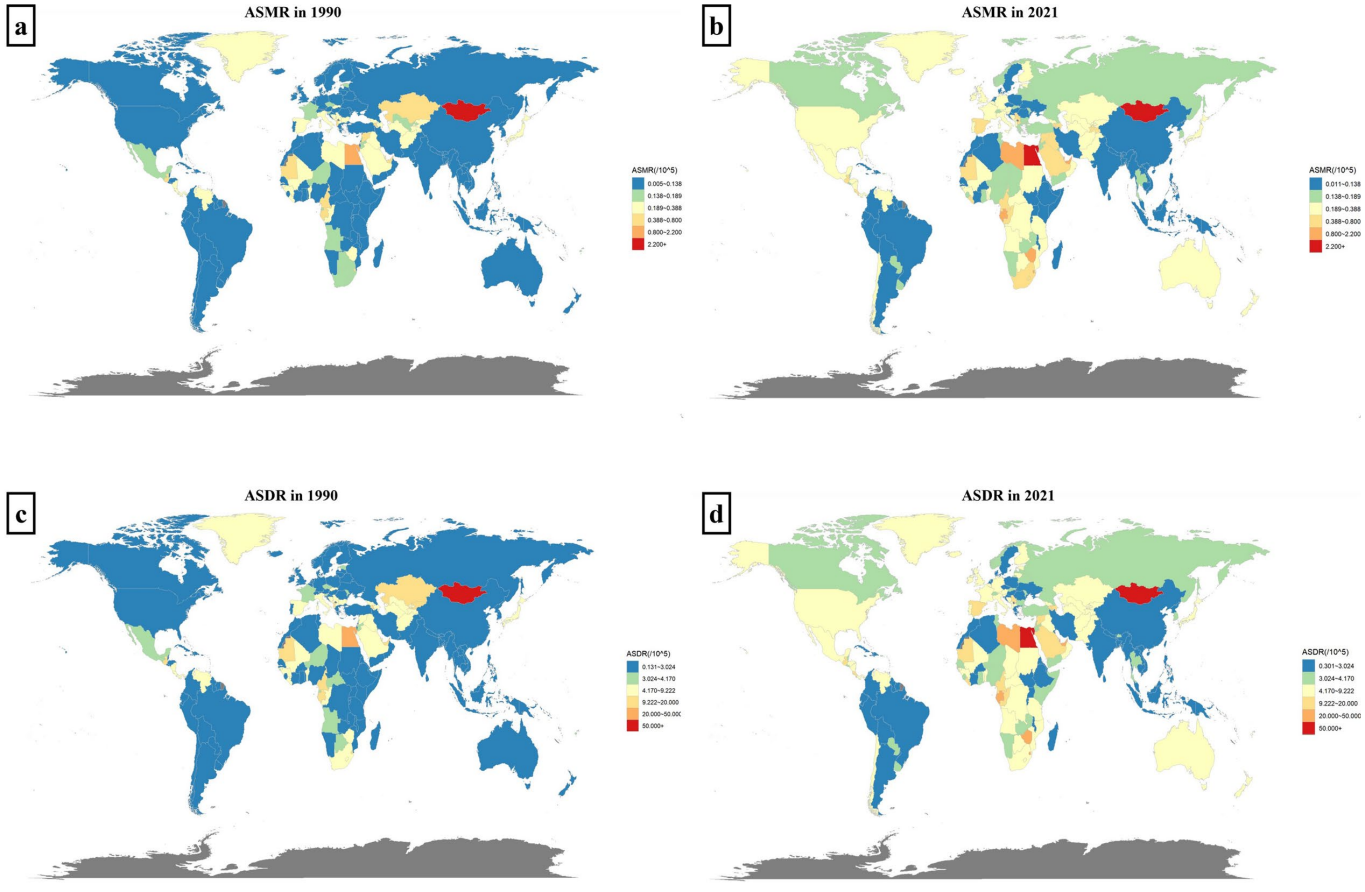
The distribution map of HCV burden related to HBMI. Panels **(a,b)** show the distributions of ASMR across regions and 204 countries in 1990 and 2021, respectively. Panels **(c,d)** represent the distributions of ASDR in 1990 and 2021, respectively. Different levels of disease burden are distinguished by colors, as indicated in the legend.

Figure 1 illustrates that in 2021, the HCV burden linked to HBMI increased compared to 1990 in most countries across Africa, North America, Oceania, Northern Europe, and North Asia. Africa bears a particularly heavy burden, with Egypt experiencing the highest impact. Notably, Mongolia faces the most significant burden among the 204 countries analyzed. Conversely, East Asia, South Asia, and South America experience a relatively lighter burden (Figure 1).

### 3.3 Sex-age distribution and global trends of hepatitis C burden associated with HBMI

**Figure 2:**
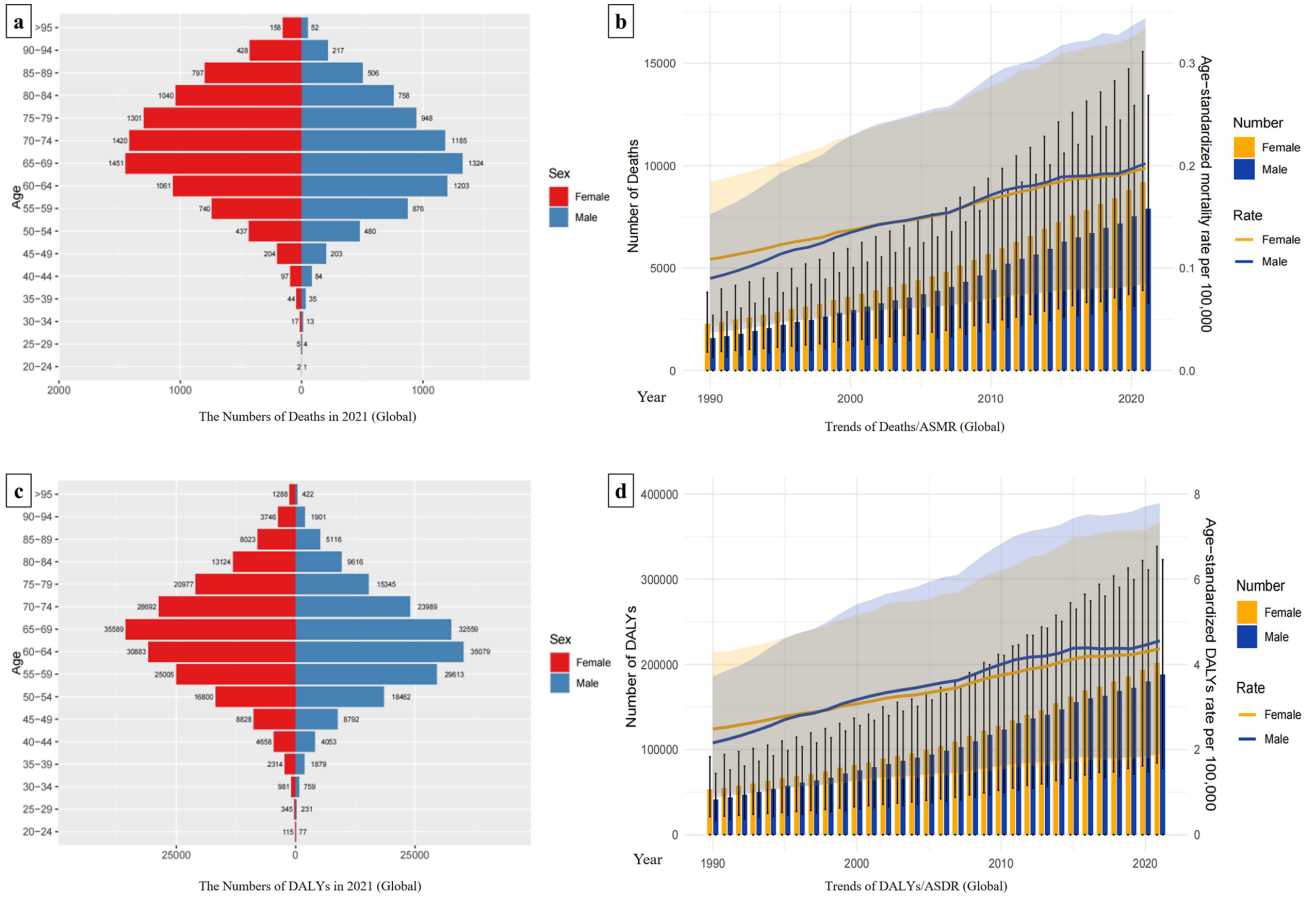
Trends in the global burden of hepatitis C associated with HBMI and its distribution by sex and age. Panels **(a,c)** show the distributions of the numbers of deaths and DALYs by sex and age globally. Women are shown in red and men in blue. In panels **(b,d)**, the bar chart represents the actual number of deaths/DALYs for males and females globally in each year. The line chart shows the changing trends of ASMR/ASDR for males and females from 1990 to 2021. Females are represented in yellow, and males are represented in blue.

The disease burden of hepatitis C linked to HBMI varies by age and gender. Globally, females experience a higher burden than males, with 9,202 deaths and 201,368 DALYs in 2021, surpassing the male figures of 7,889 deaths and 187,893 DALYs. For females, deaths and DALYs peak at ages 65–69. In contrast, male deaths peak at ages 65–69, while DALYs peak at ages 60–64, indicating a slightly younger onset of burden in males compared to females (Figures 2a,c).

The global ASMR and ASDR consistently increased annually across different genders from 1990 to 2021. Since 2000, there has been a convergence in ASMRs between males and females, whereas since 1997, ASDRs for males have surpassed those for females. Additionally, throughout the period 1990 to 2021, the total number of Deaths and DALYs have consistently been higher for women compared to men (Figures 2b,d).

Besides, we also analyzed the sex-age distribution of hepatitis C burden linked to HBMI in different regions. In Low-middle SDI regions, female deaths totaled 1,508 and DALYs were 39,450, both lower than male figures (Deaths: 1,730; DALYs: 47,051). In High SDI regions, female deaths reached 2,924 with DALYs at 55,296, also lower than males (Deaths: 3,183; DALYs: 68,962). Conversely, in other SDI regions, females experienced a greater disease burden than males. The age peaks for Deaths and DALYs varied slightly by regions, predominantly occurring in the 60–69 age group. In each region, males reached the burden peak slightly younger than females. Notably, in High SDI regions, female deaths peaked in the 75–79 age group, whereas male deaths peaked in the 70–74 age group (Supplement 2 in Supplementary material).

### 3.4 Comparison of hepatitis C burden associated with HBMI among regions and their Joinpoint regression analysis

**Figure 3:**
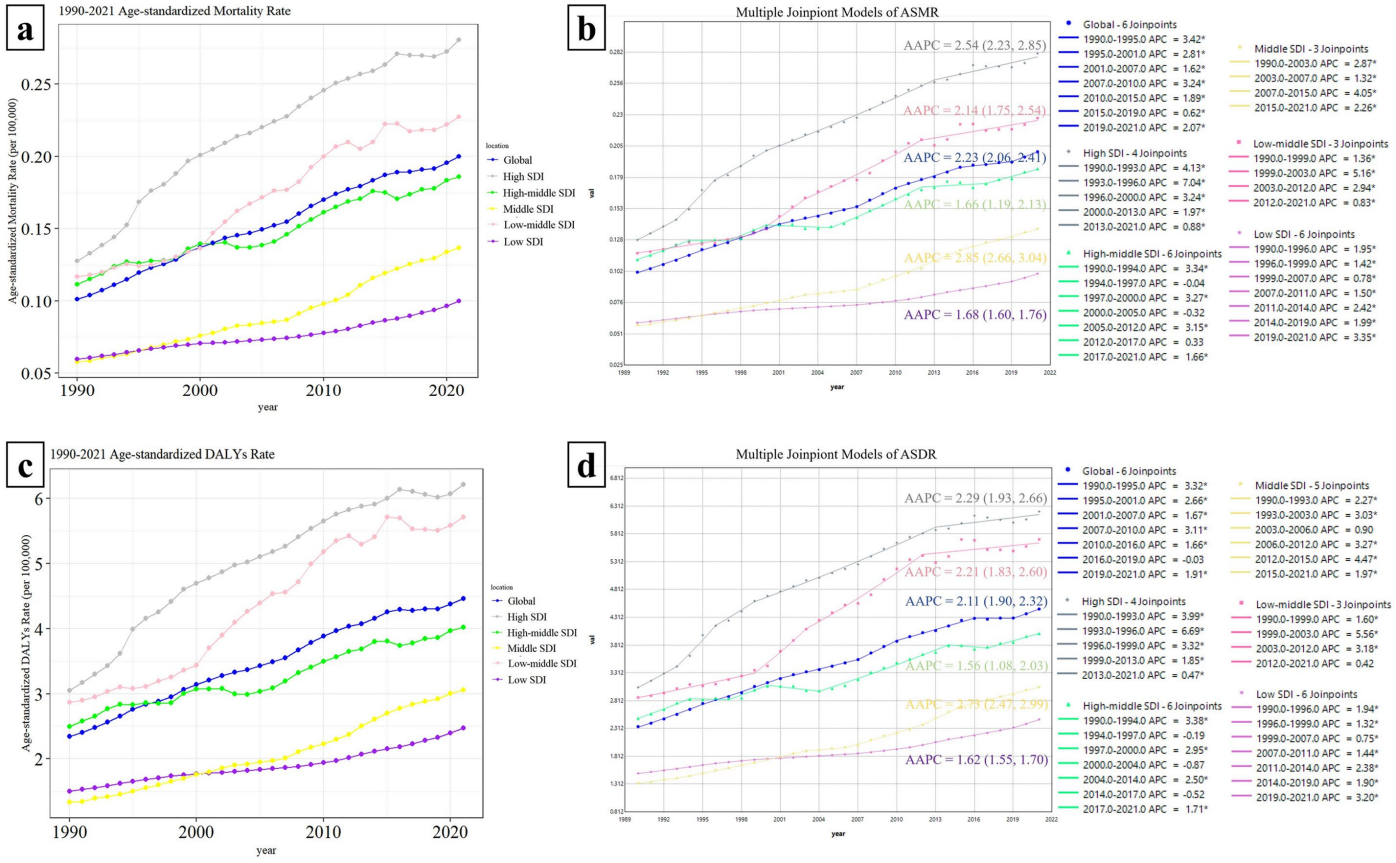
Comparison of Hepatitis C Burden Associated with HBMI among regions and their Joinpoint Regression Analysis. Panels **(a,c)** compares the changing trends of ASMR/ASDR between the globe and each SDI region. The line graphs of different regions are represented by different colors. Panels **(b,d)** shows the Joinpoint regression analysis of ASMR and ASDR in each region. In the change curves of different regions, the years with similar change trends were grouped into the same time period. According to the fitting effect, the change curves of each region could be divided into different time periods with statistical differences. A larger APC value indicates a faster upward trend during that time period, and a negative APC value indicates a downward trend during that time period. The average change trend over the entire 30-year period is represented by AAPC. If the 95% CI of the corresponding AAPC estimate is >0, the ASR index shows an upward trend; if the 95% CI < 0, it shows a downward trend; if it includes 0, it shows a stable trend.

Over the past 30 years, the ASMR and ASDR have gradually increased over time across various regions. High SDI regions bear the heaviest disease burden, consistently exhibiting significantly higher ASMR and ASDR than other SDI regions. The upward trend of the disease burden in Low-middle SDI regions and Middle SDI regions has accelerated significantly since 2000. Moreover, in Low-middle SDI regions, annual ASMR and ASDR are second only to those in High SDI regions. Among all regions, Low SDI regions experience the lowest burden (Figures 3a,c).

Joinpoint regression analysis revealed a significant upward trend in both the ASMR and ASDR across all regions from 1990 to 2021, with AAPC values and 95% CI exceeding 0. The Middle SDI region showed the most prominent increasing trend, with the AAPC values of ASMR and ASDR being 2.85 (2.66, 3.04) and 2.73 (2.47, 2.99) respectively. The High SDI region followed, with AAPC values of 2.54 (2.23, 2.85) for ASMR and 2.29 (1.93, 2.66) for ASDR. The Low-Middle SDI region ranked third with AAPC values of 2.14 (1.75, 2.54) and 2.21 (1.83, 2.60), respectively. The specific time periods with the fastest-growing burden in each region are shown in the figure (the largest APC value indicates the fastest upward trend) (Figures 3b,d).

### 3.5 The relationship between the disease burden of HBMI-related HCV and SDI values

According to the classification of SDI regions outlined in Section 2.2 and the most recent SDI values for individual countries, it is evident that in 2021, there are 45 countries classified as High SDI (SDI ≥ 0.805129), 48 as High-middle SDI (0.689504 ≤ SDI < 0.805129), 36 as Middle SDI (0.607679 ≤ SDI < 0.689504), 37 as Low-middle SDI (0.454743 ≤ SDI < 0.607679), and 33 as Low SDI (SDI < 0.454743) (The SDI values of each country in 2021 can be found in the Supplementary Data Sheet).

**Figure 4:**
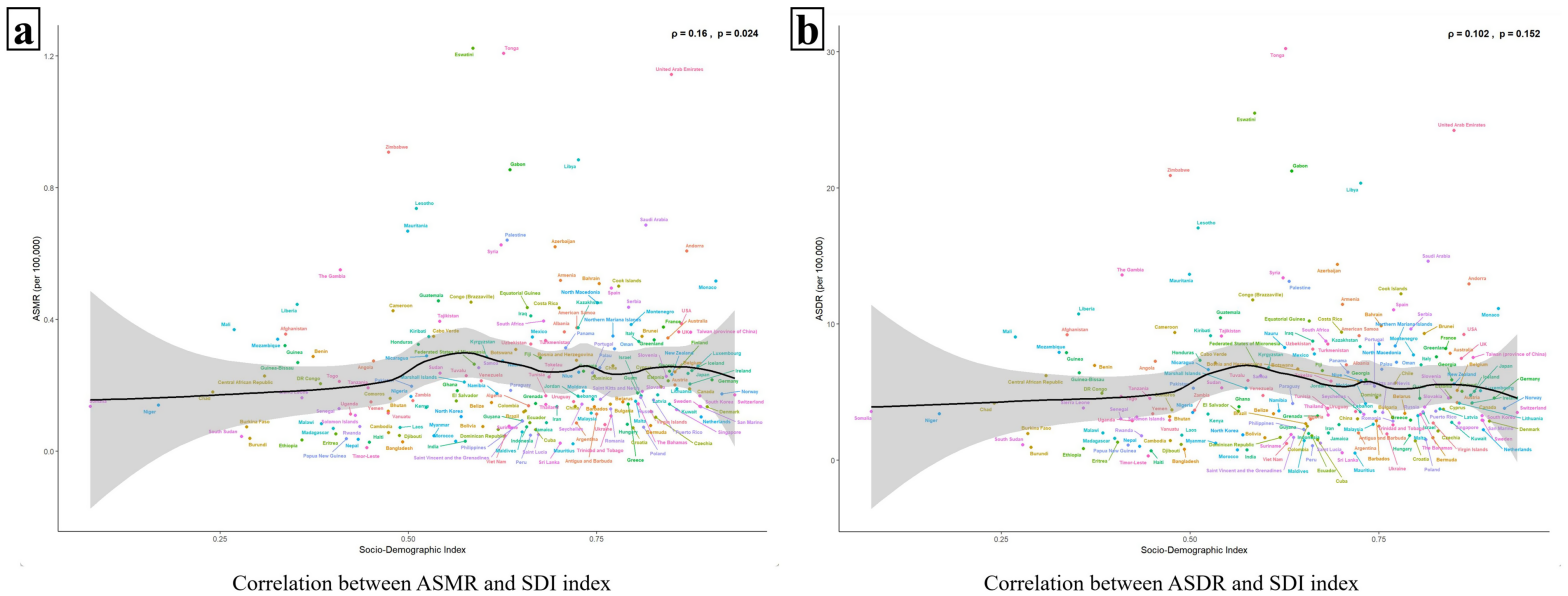
The relationship between ASMR/ASDR and 204 countries with different SDI values. Panels **(a,b)** show the relationships between ASMR/ASDR and SDI values, respectively. The black solid line represents the fitted curve based on the data of 204 countries with different SDI values and their corresponding ASMR/ASDR values, and the gray area indicates its confidence interval. The *p* value < 0.05 was considered statistically significant.

In Figure 4a, a correlation is observed between ASMR and SDI values (*p* = 0.024 < 0.05), displaying a similar “double-peak” pattern. When the SDI value is less than 0.50, ASMR increases very slowly or even remains flat as the SDI value rises. Notably, for SDI values exceeding 0.5, ASMR exhibits accelerated growth. At an SDI value of approximately 0.6, ASMR reaches a minor peak and then gradually declining. Subsequently, ASMR rises again as the SDI value nears 0.75, forming a second minor peak. Beyond an SDI value of 0.8, ASMR predominantly maintains a plateau akin to the second peak. In addition, the relationship curve between ASDR and SDI, as depicted in Figure 4b, also mirrors the “double-peak” trend observed in Figure 4a; however, no significant correlation is statistically supported between them (*p* = 0.152 > 0.05) (Figure 4).

**Figure 5:**
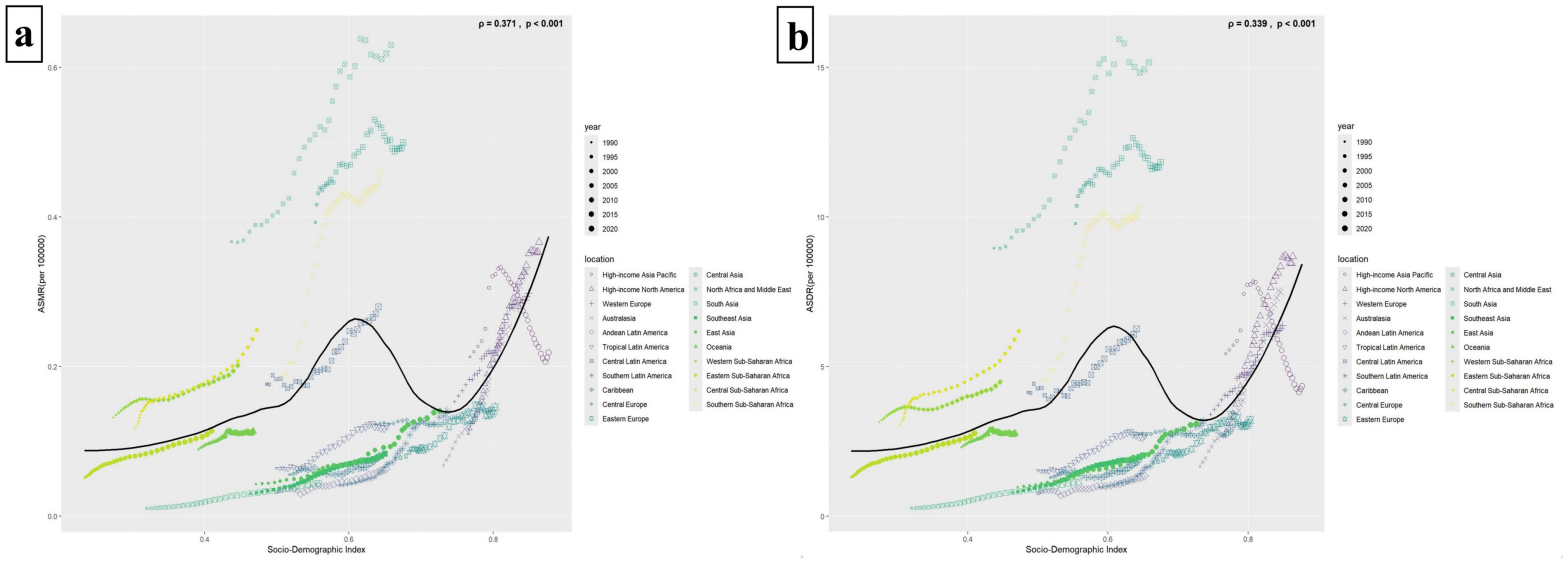
The relationship between ASMR/ASDR and 21 GBD super regions with different SDI values. Panels **(a,b)** illustrate the relationships between ASMR/ASDR and 21 regions with varying SDI values. The solid black line depicts the fitted curve derived from the data of these 21 regions and their respective ASMR/ASDR. A *p*-value of less than 0.05 signifies a statistically significant difference. Different regions are represented by different icons. The icons from small to large represent the temporal trends from 1990 to 2021.

Figures 5a,b illustrate that both the ASMR and the ASDR are correlated with the SDI values of the 21 GBD regions (ρ_ASMR_ = 0.371, *p* < 0.001, ρ_ASDR_ = 0.339, *p* < 0.001). Similar to Figure 4 above, the ASMR and ASDR reached their first minor peaks in regions with an SDI value of around 0.6 (e.g., North Africa and Middle East, Central Asia, Southern Sub-Saharan Africa, Central Latin America). Subsequently, when the SDI value was greater than 0.75, the ASMR and ASDR increased again. For example, in High-income North America, High-income Asia Pacific, Australasia, and Western Europe. Among all 21 regions, the disease burden has generally increased since 1990, except in the High-income Asia Pacific, where it has decreased (Figure 5).

### 3.6 Age-period-cohort model of HCV mortality rate associated with HBMI

**Figure 6:**
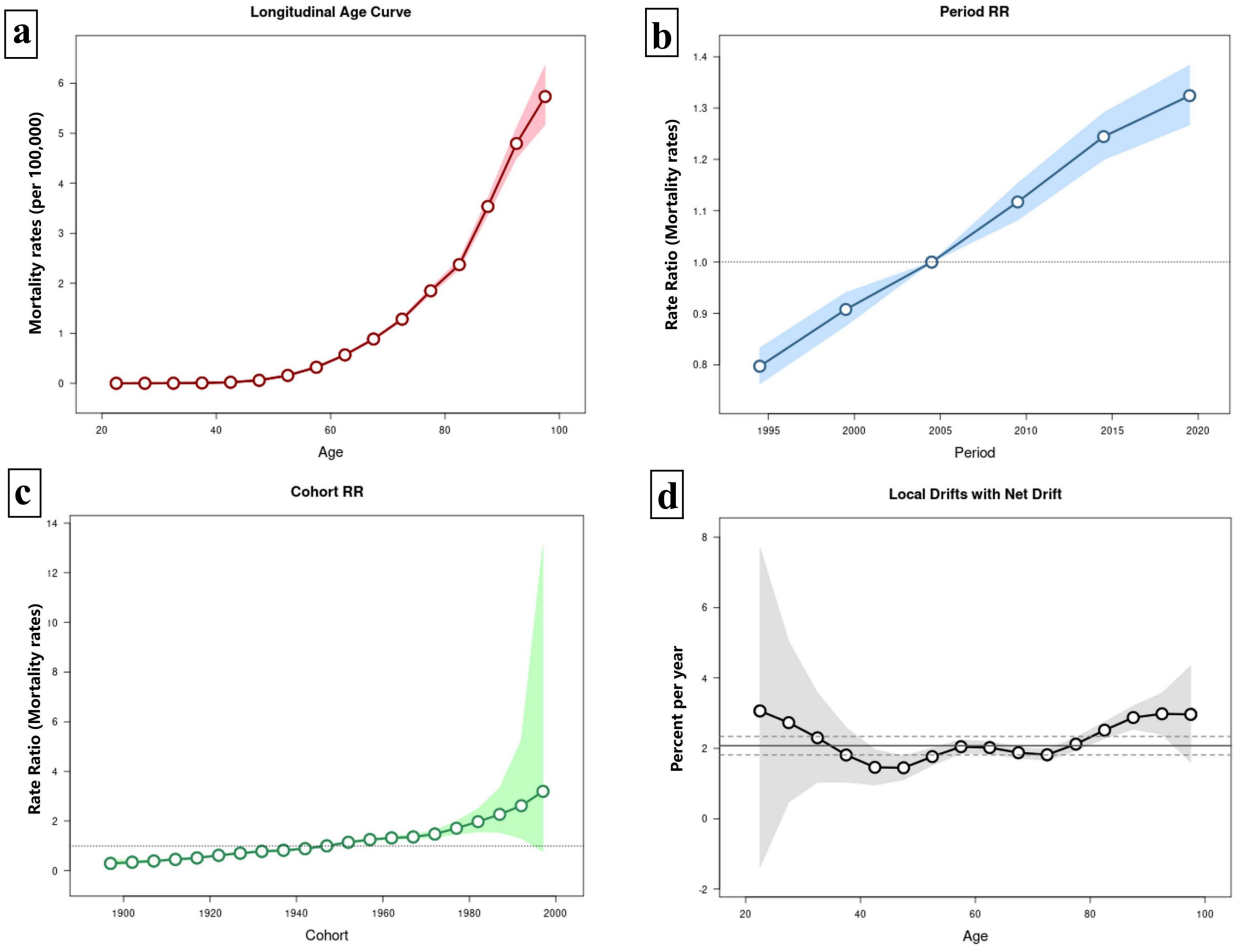
Age-period-cohort (APC) model of HCV mortality rate associated with HBMI. Panel **(a)** illustrates the age effect on HCV mortality linked to HBMI, while panel **(b)** depicts the period effect, and panel **(c)** shows the cohort effect. Panel **(d)** presents the local drift of mortality for each age group (i.e., the annual percentage change in mortality over time). A value above zero signifies an upward trend in mortality for that age group, and vice versa. The central solid black line represents the net drift, reflecting the overall drift scenario.

Figure 6 illustrates the APC model for HCV mortality linked to HBMI. Figure 6a displays the net age effect after adjusting for period and cohort influences. In younger populations (20–40 years old), the mortality rate remains low and relatively stable. However, after age 50, the mortality rate increases sharply, and the risk of death escalates with age. The period model, adjusted for age and cohort effects, reveals that HCV mortality associated with HBMI rises annually with no indication of decline. In the birth-cohort model, although the curve appears flat, it exhibits an overall upward trend. Across the included age range, the local drift is consistently positive, indicating an upward trend in HCV mortality associated with HBMI over time in each age group. Overall, the net drift is 2.074% (1.812, 2.338%) (Figure 6).

### 3.7 Decomposition analysis of HCV burden associated with HBMI in the world and each SDI region from 1990 to 2021

**Figure 7:**
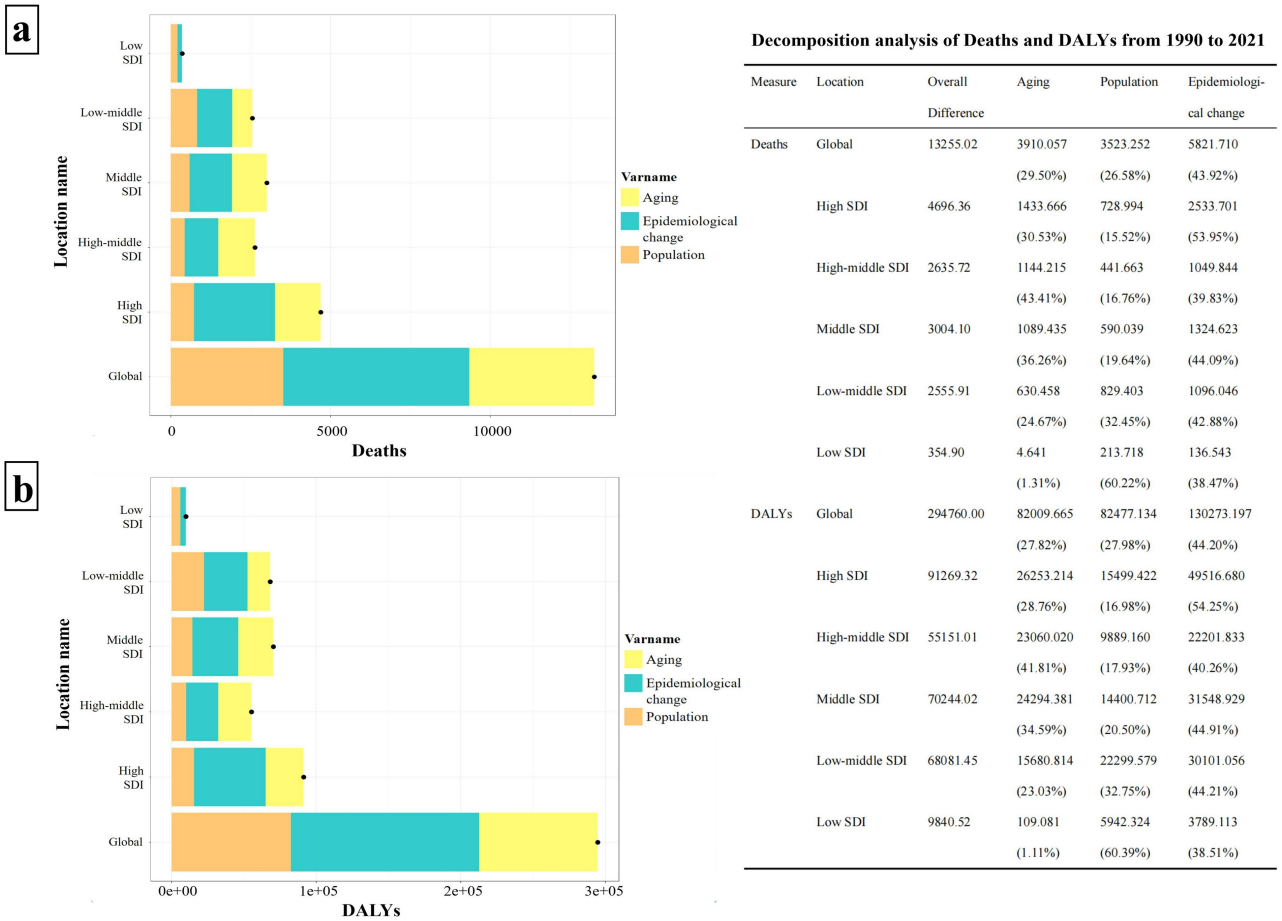
Decomposition analysis of HCV burden associated with HBMI in the world and each SDI region from 1990 to 2021. Panel **(a)** illustrates the decomposition analysis of HCV deaths linked to HBMI, while panel **(b)** depicts the decomposition analysis of DALYS. The bar charts, displayed in three distinct colors, represent the components of population growth, aging, and epidemiological changes. Black dots signify the overall change values contributed by all three components combined. For each component, a positive value’s magnitude indicates an increase in the disease burden attributed to that component, whereas a negative value’s magnitude signifies a decrease. The chart on the right details the specific contribution values of each component across various regions.

Over the past 30 years, the disease burden of HCV associated with HBMI has significantly increased globally and in each SDI region. Notably, the High SDI region experienced the largest increases in both deaths and DALYs, with aging contributing 30.53 and 28.76%, respectively, and population growth accounting for 15.52 and 16.98%. The Middle SDI region saw the second-largest increases, with aging contributing 36.26% to deaths and 34.59% to DALYs, while population growth accounted for 19.64 and 20.50%. The influence of aging seems to escalate with the rise of SDI values, peaking in the High-middle SDI region (Deaths: 43.41%, DALYs: 41.81%). Conversely, the impact of population growth tends to increase as SDI values decline, with the Low SDI region showing the highest contribution (Deaths: 60.22%, DALYs: 60.39%). Despite the significant effects of aging and population growth on the HBMI-related HCV burden, the primary global contributor remains epidemiological change (Deaths: 43.92%, DALYs: 44.20%) (Figure 7).

### 3.8 Frontier analysis of ASMR and ASDR in countries with different SDI levels

**Figure 8:**
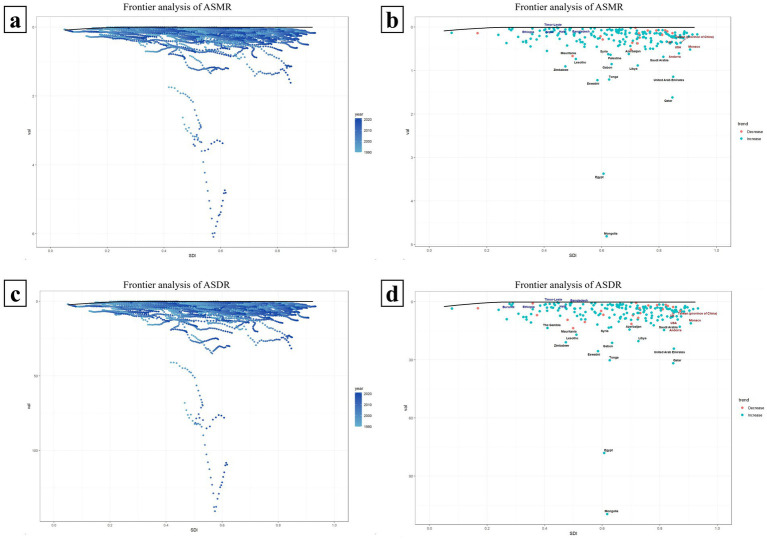
Frontier analysis of ASMR and ASDR in countries with different SDI levels. Panels **(a,c)** respectively illustrate the trends of the frontiers of ASMR and ASDR from 1990 to 2021 among 204 countries with different SDIS. The farther a country is from the solid black frontier line, the greater the effective difference, indicating that the country has more room for improvement. Panels **(b,d)** show the frontier analysis of ASMR and ASDR in each SDI country based on the data in 2021. Countries are represented by dots, where green dots indicate an increase in ASMR or ASDR compared to 1990, and red dots indicate a decrease compared to 1990.

Figure 8 presents a frontier analysis of the ASMR and ASDR across countries with varying SDI values. The frontier is depicted by a solid black line. Figures 8a,c specifically illustrate the trends in ASMR and ASDR frontier changes from 1990 to 2021. The data reveal that in most countries, the effective difference widens annually, suggesting an increasing potential to reduce the HBMI-associated hepatitis C burden over time. Concurrently, as time goes by, the SDI values of each country also rise annually, and the ASMR and ASDR of each country also show an increasing effective difference as their own SDI values rise annually (Figures 8a,c).

Figures 8b,d illustrate a frontier analysis of the ASMR and ASDR across countries with varying SDI levels in 2021. The frontier is depicted by a solid black line, with countries represented by dots. Green dots indicate an increase in ASMR or ASDR compared to 1990, while red dots signify a decrease. As shown in the figures, the ASMR or ASDR has increased in most countries since 1990. The top 15 countries with the largest effective differences (i.e., the greatest gaps between their ASDR/ASMR and the frontier) are marked in black, including Mongolia, Egypt, Qatar, Eswatini, Tonga, United Arab Emirates and so on. Frontier countries with SDI values less than 0.5 and minimal effective differences are marked in blue, such as Ethiopia, Haiti, and Timor-Leste, which could serve as model countries. Countries with SDI values greater than 0.85 and relatively high effective differences, despite their development, are marked in red and include the UK, United States, Andorra, Monaco (Figures 8b,d).

### 3.9 ARIMA prediction models

**Figure 9:**
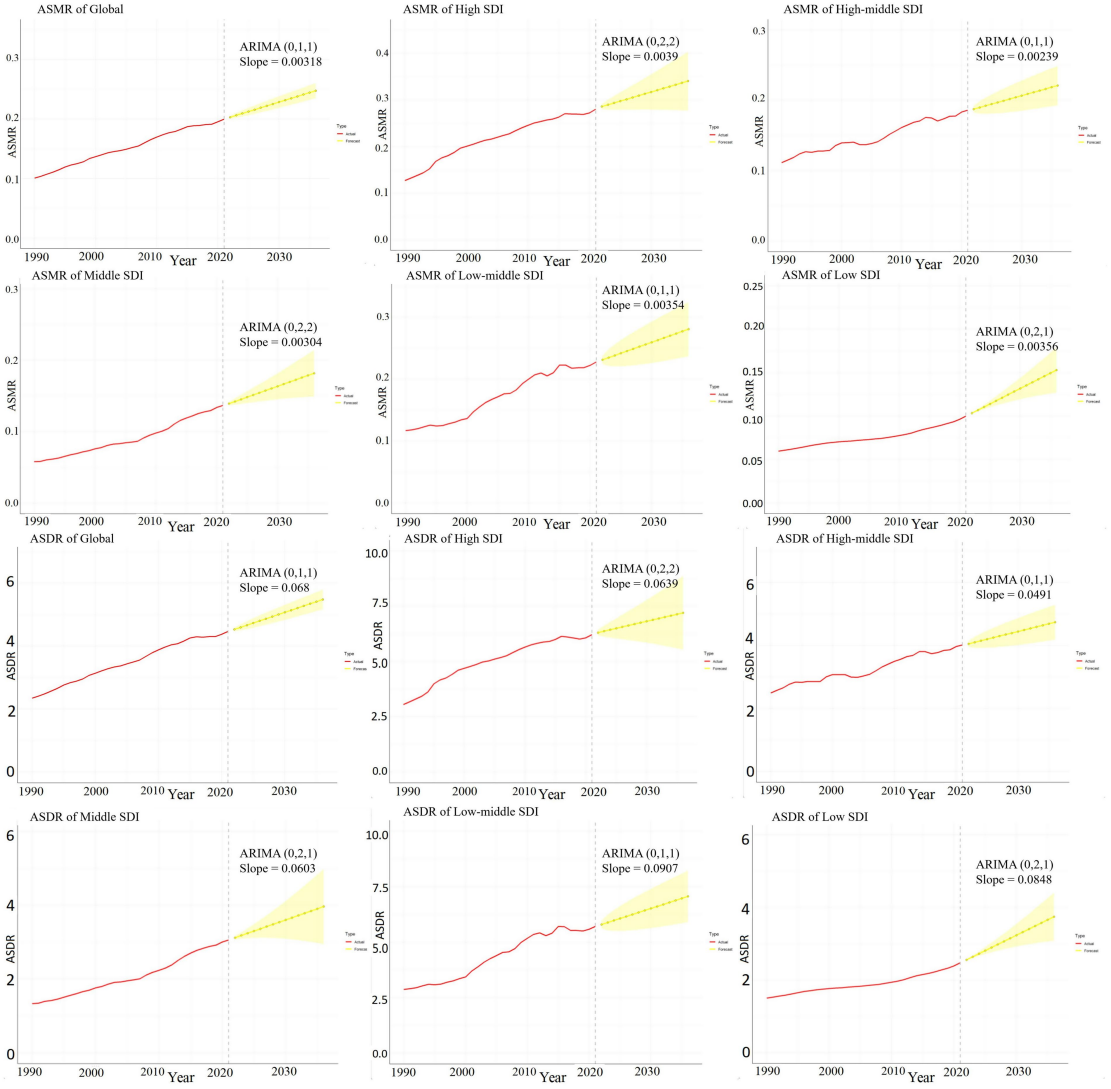
ARIMA predictive model of hepatitis C burden associated with HBMI. This figure quantitatively depicts the trends of ASMR and ASDR related to hepatitis C associated with HBMI in the next 15 years globally and in each SDI region using the ARMIA prediction model. The red line represents the actual trends of ASMR and ASDR from 1990 to 2021, and the yellow dashed line and shaded area represent the predicted trends and their 95% CIs. The model is expressed as ARIMA (p, d, q) and “Slope” indicates the slope of the predicted curve.

Figure 9 illustrate the projected trends in HCV-related ASMR and ASDR associated with HBMI over the next 15 years, both globally and across each SDI region, using the ARMIA prediction model. The red line indicates actual ASMR and ASDR trends from 1990 to 2021, while the yellow dashed line and shaded area show predicted trends with 95% CI. The analysis predicts a continued increase in ASMR and ASDR worldwide and in each SDI region, underscoring the escalating impact of obesity on the hepatitis C disease burden without effective interventions. Among the SDI regions, the High SDI region is expected to have the fastest growth in ASMR in the next 15 years (the average slope of the forecast curve is 0.0039). Its ASMR is projected to increase from 0.28 per 100,000 people in 2021 to 0.34 per 100,000 people in 2036. The fastest growth in ASDR over the next 15 years is in the Low-middle SDI region (average slope of the forecast curve is 0.0907). Its ASDR is projected to increase from 5.71 years per 100,000 people in 2021 to 7.07 years in 2036 (Figure 9).

Supplements 3, 4 in Supplementary material used the data on the HCV disease burden associated with HBMI in 2010 to predict the ASMR and ASDR for the next 11 years. After comparing with the actual results from 2011 to 2021, it was found that there were statistical differences between the predicted and actual results in the High SDI, Middle SDI, and Low - middle SDI regions (*p* < 0.05). However, no statistical differences were observed at the global level, in the Low SDI, and High - middle SDI regions (*p* > 0.05) (Supplements 3, 4 in Supplementary material).

## 4 Discussion

Hepatitis C continues to threaten public health significantly. With the application of pan-genotypic direct-acting antivirals (DAAs), the improvement of HCV screening and diagnosis, the effective control of transmission routes, and the development of hepatitis C virus vaccines, etc., the incidence and mortality of hepatitis C seem to have decreased (26–28). However, the coverage of prevention, diagnosis, and treatment of hepatitis C remains inadequate (27). Many patients struggle with early detection, diagnosis, and treatment, leading to a persistent rise in the overall number of deaths from hepatitis C in the past 30 years (18, 29). Statistics indicate that intravenous drug use is the leading cause of death from liver cancer related to hepatitis C (Supplement 1 in Supplementary material). Previously, most studies concentrated primarily on the effects of intravenous drug use on hepatitis C. From 2015 to 2021, the annual incidence of new HCV infections attributed to injecting drug use was estimated at 833,760 cases (30). Besides, smoking and alcohol consumption are also significant risk factors for hepatitis C-related deaths, as they are recognized hepatotoxins that contribute to chronic liver diseases and liver cancer (31, 32). Notably, obesity is currently considered to be closely associated with the prognosis of patients infected with HCV (9–11). Research indicates that individuals with hepatitis C virus infection and cirrhosis face a significantly elevated risk of developing cancer when their BMI exceeds 35 (with a hazard ratio (HR) reaching 4.52) (33, 34). According to GBD statistics, as of 2021, obesity ranks second in risk for hepatitis C-related liver cancer deaths, surpassing smoking and drinking, and trailing only drug use (35) (Supplement 1 in Supplementary material). Meanwhile, our study indicates a yearly increase in hepatitis C-related deaths associated with obesity. Thus, to meet the goal of eliminating hepatitis C by 2030, managing body weight is crucial.

The pathophysiological mechanisms between HCV virus and lipids can be divided into two parts key aspects. On the one hand, it is the impact of the virus on lipid metabolism, mainly including: (1) HCV can activate multiple lipid transcription factors and stimulate the synthesis of *de novo* fat to promote the assembly of HCV lipid virus particles (12); (2) To provide raw materials for virus replication, HCV can enhance the synthesis of phospholipids, sphingomyelin, and lipotoxic ceramide (36); (3) HCV can inhibit mitochondrial fatty acid oxidation (37); (4) HCV can disrupt the assembly of VLDL and interrupt its secretion pathway (38); (5) Upregulate the pentose phosphate pathway to affect lipid metabolism (12). The above impacts of HCV on lipid metabolism can ultimately lead to hepatic fat accumulation and hepatic steatosis (39). Moreover, studies by Rubbia-Brandt et al. (40) and Poynard et al. (41) have indicated that even without metabolic risk factors associated with NAFLD, different HCV genotypes (especially genotype 3) and HCV RNA viral loads are significantly associated with higher steatosis scores.

On the other hand, the effects of fat on HCV patients are mainly manifested in the following aspects: (1) The accumulation of lipids in the liver leads to lipotoxicity, causing hepatocyte death and activating the chronic wound - healing response, which may rapidly progress to fibrosis and increase the risk of HCC (12, 42). (2) Hepatocyte steatosis occurs in patients infected with the HCV virus, resulting in metabolic disorders of related cytokines (such as a significant increase in visfatin levels) and accelerating the development of HCC (43). (3) Obesity triggers systemic low - grade inflammation in the body, accelerating the development of liver cirrhosis and liver cancer (44). (4) Obesity is associated with a reduced clearance rate of the hepatitis C virus (11). In summary, the HCV virus can disrupt the host’s lipid metabolism, leading to visceral fat accumulation. In obese or overweight HCV patients, fat accumulation can be exacerbated, further resulting in lipotoxicity. This forms a vicious cycle, accelerating the occurrence of liver fibrosis and progressing to HCC. This highlights the importance of weight loss in the treatment of hepatitis C.

Utilizing the GBD 2021 database, we conducted a comprehensive evaluation of hepatitis C-related mortality and DALYs, with HBMI as a risk factor, over the past three decades. We found that globally, the burden of HCV associated with obesity increased over time. Total Deaths and DALYs increased significantly compared with 1900, and ASMR and ASDR showed a significant upward trend (EAPCs = 2.20 and 2.10, respectively). This differs from the findings of other studies on the burden of hepatitis C disease, which suggest that although the number of hepatitis C deaths is increasing annually, the mortality rate is decreasing (29). Because these studies is based on all factors influencing the burden of hepatitis C disease. However, when only focusing on the impact of obesity in hepatitis C, we found that the mortality rate, number of deaths, DALY, and ASDR have all shown an upward trend over the past three decades. In our APC model, after adjusting for the effects of age and birth cohort, the mortality rate of HCV linked to HBMI demonstrated a significant upward trend over time periods. Furthermore, results from the local drift model indicated that mortality rates across all age groups also increased over time. This trend is likely linked to the contemporary obesity epidemic. The reason is that the prevalence of obesity has been continuously rising across all countries or regions, genders, and age groups worldwide (15). As the liver serves as a metabolic factory for fat, the uncontrolled increase in the global obesity rate will inevitably exacerbate the burden of hepatitis C disease.

The highest number of HCV-related deaths and DALYs linked to obesity is found in individuals aged 60 to 69. However, according to the APC model, mortality rates increase exponentially with age. The reason is that the population over 70 years old is smaller than that in the 60–69 age group, resulting in a relatively lower number of deaths but a higher rate. Therefore, the burden of HBMI on HCV patients gradually intensifies with age. Studies identify age as an independent risk factor for liver cancer, and the increase in age is directly related to the incidence of liver cancer (45). Statistical analyses reveal a marked increase in liver cirrhosis deaths post 20 years old, peaking at ages 60–64 for men and 65–69 for women (46). In addition, some epidemiological surveys across various countries and regions indicate a concurrent rise in obesity and an aging population (15). And their projections suggest that by 2050, nearly 25% of the global obese population will be over 65 years old (15). In conclusion, given the high incidence of liver cancer and liver cirrhosis in the older adults, obesity further exacerbates the burden on the liver, thereby accelerating HCV-related deaths.

Additionally, although the impact of birth cohort on obesity-related HCV mortality is relatively minor, it does exhibit a gradual increase over time. This trend is not only linked to the widespread adoption of HCV screening but also to mother-to-child transmission, which merits attention. Statistics indicate that the HCV infection rate among pregnant women has risen over time (47). By 2019, the number of HCV-infected women of childbearing age (15–49 years old) worldwide reached 14.68 million, representing 21% of the virus infection rate (48). Consequently, the risk of mother-to-child HCV transmission has also grown. Therefore, women of childbearing age should be prioritized in efforts to prevent hepatitis C virus, and universal prenatal screening is viewed as an effective strategy to prevent vertical transmission in future pregnancies (48).

The HCV burden linked to obesity exhibits gender differences, with females more impacted than males. Over the past 30 years, the global total of female deaths and DALYs has consistently surpassed that of males annually. Previous epidemiological data have shown that the overall burden of hepatitis C is greater in males than in females due to higher rates of drug use, smoking and alcohol consumption in men than in women (49). However, in terms of obesity alone, the HCV burden in females is higher than that in males. This may be related to gender-specific nutritional and physical activity patterns, estrogen’s role in promoting subcutaneous fat, reproductive factors, and hormonal changes during pregnancy and menopause, which lead to a higher prevalence of obesity in females than in males (15, 50–52). Consequently, female hepatitis C patients are more affected by obesity. Therefore, female patients infected with the HCV virus should pay more attention to biochemical interventions for weight loss, such as low-fat diets, low-carbohydrate diets, low-energy diets, and organized physical activity interventions (53). When lifestyle interventions are ineffective, drug interventions such as GLP-1 receptor agonists can be further considered (54).

The ASMR and ASDR in each SDI region have consistently risen in the past 30 years (the EAPC in each region is greater than 0). Obesity rates have been rising across various SDI regions, which indirectly impacts the obesity-related hepatitis C mortality and ASDR in these areas (12, 15). Notably, the High SDI region reports the highest annual total Deaths and total DALYs, and also the highest ASMR and ASDR, indicating that obesity imposes the greatest burden of hepatitis C in this area. The persistence of high obesity rates in developed areas can be linked to factors such as the positive correlation between economic status and dietary patterns characterized by high caloric, sugary, and fatty foods, sedentary lifestyles with limited physical activity, and elevated levels of psychological stress (15, 55).

However, we also found that the ASMR and ASDR in the Middle SDI region have increased most significantly over the past 30 years (the AAPCs were 2.85 and 2.73, respectively). Moreover, Low-middle SDI regions rank second in ASMR and ASDR annually, following High SDI regions. These findings highlight obesity’s escalating impact on the hepatitis C disease burden in these areas. Middle and low-middle SDI regions have undergone rapid economic development in recent decades, prompting notable shifts in dietary habits and lifestyles. Meanwhile, inadequate healthcare services in these areas have hindered effective management of metabolic diseases and obesity interventions, leading to a swift rise in overweight and obesity prevalence (55–57). This escalating obesity trend also further intensify the burden of hepatitis C.

In examining the relationship between obesity-related HCV burden and sociodemographic development, we found that the peaks of the disease burden were mainly concentrated in countries and regions with an SDI value around 0.6 and above 0.75. This observation aligns with the above findings, indicating that the disease burden mainly affects Middle SDI and High SDI regions. From the national map, these regions include North Africa and Middle East, Central Asia, High-income North America, Australasia, Western Europe, etc. Notably, Mongolia and Egypt experience the most significant burden at the national level.

The generally severe HCV burden in Africa primarily stems from inadequate medical and health conditions, including issues with blood safety, hygiene in medical injections, and HCV screening and treatment (58). Contributing factors also include population growth and drug abuse (58, 59). Consequently, the overall prevalence of HCV in Africa is more severe than in other regions. Given the large number of people with hepatitis C, and considering that obesity can worsen the prognosis of chronic hepatitis C, this will inevitably have a significant impact on the HCV burden in this region. Furthermore, Egypt and Mongolia, classified as Middle SDI countries, experience the highest HCV burden related to HBMI. This is because the rates of patients infected with the hepatitis C virus in these two countries is relatively high (4.61 and 6% respectively), far exceeding the global average, and chronic hepatitis C is the main cause of liver cirrhosis and liver cancer in these areas (60, 61).

In North America and Oceania, despite the increased availability of HCV diagnosis and DAA treatment, HCV remains prevalent due to the relatively high rate of intravenous drug use (62, 63). Additionally, the high level of development in these regions has led to a high-calorie diet structure, contributing to rising obesity rates (15). Therefore, HBMI also contributes significantly to the HCV burden in these regions. Hence, different measures should be taken to address the HCV burden associated with HBMI in different regions. For instance, in Africa, the priority should be enhancing medical and health services, whereas in developed areas, efforts should focus on promoting weight loss and controlling transmission routes, such as drug use, tattooing, and sexual transmission.

According to the decomposition analysis model, compared with 1990, the impacts of aging and population growth to the disease burden of HBMI-related HCV cannot be ignored. The contribution of aging appears to increase with the rise of the SDI value, while the contribution of population growth seems to increase with the decline of the SDI value. Social development levels are correlated with fertility rates. Economic level, traditional concepts, cost of raising children, social security, living environment, child mortality rate, etc., are all factors contributing to the relatively higher fertility rate in low-development regions, resulting in a significantly larger population in these regions than in high-development regions. However, poverty and inadequate healthcare in these low-development regions lead to higher rates of premature death, thereby diminishing the impact of aging on the HBMI-related HCV disease burden. Conversely, factors such as high parenting costs, social pressures, and low employment rates contribute to low fertility rates in highly developed regions. Yet, advancements in medical and health services, along with high quality of life, result in longer average life expectancies in these areas. Consequently, the impact of population growth and aging on the disease burden of HBMI-related HCV varies significantly between High SDI and Low SDI regions. Nevertheless, globally, the epidemiological shifts in HBMI-related HCV are the primary contributors to the disease burden. And the proportion of epidemiological changes in each SDI region cannot be ignored. As mentioned above, this is related to the relatively backward medical and health levels in less developed regions and the high-calorie diet and high drug use in highly developed regions.

In terms of the frontier analysis of obesity-related HCV burden, most countries currently have significant potential for improvement, and they are getting farther away from the frontier target as the years progress. Notably, Egypt and Mongolia bear the highest frontier burdens. The above indicates that the current HCV burden related to HBMI has already affected most countries, so timely measures should be taken to alleviate this trend. For instance, we can still take some countries with relatively small frontier burden as models, such as Ethiopia, Haiti, Timor-Leste, etc. Their dietary structures, living patterns, and relevant control measures for patients infected with hepatitis C are worthy of our reference.

Prognostic models for the next 15 years predict an annual increase in the disease burden associated with HBMI globally and across all SDI regions, with the fastest growth in High-SDI and Low-middle SDI areas. Results from the decomposition analysis suggest that epidemiological changes, population aging, and population growth are all contributing factors to the increasing burden. Therefore, to mitigate the impact of epidemiological changes, it is crucial to continue promoting HCV screening and treatment and to interrupt transmission routes. For instance, strict regulation of intravenous drug use and blood products is necessary, along with education about other potential blood-borne transmission routes like tattooing and sharing sharp tools. Additionally, sexual transmission education and measures to prevent mother-to-child transmission should be prioritized. Simultaneously, raising awareness about obesity among HCV-infected patients and improving the prognosis of chronic HCV through weight management is urgent. To address the effects of population growth and aging, regions with low development levels should focus on controlling high fertility rates and enhancing medical and health services to counteract the impacts of population growth and premature mortality on disease burden. Conversely, highly developed countries should implement policies to encourage childbearing and address population aging.

In addition, we used the 2010 data on the HCV disease burden associated with HBMI to predict the ASMR and ASDR for the next 11 years. These predictions were then compared with actual data from 2011 to 2021. The analysis revealed statistical differences between predicted and actual results in High SDI, Middle SDI, and Low-middle SDI regions (*p* < 0.05). However, these differences were not significant in practical terms. Moreover, on a global scale, no statistical difference emerged between the predicted and actual outcomes, indicating that the prediction model aligned well with reality. Returning to the prediction results for the data in 2021 in this study, the forecast of the HCV disease burden linked to HBMI for the next 15 years appears reliable and can effectively guide future intervention strategies.

### 4.1 Limitations

This study utilized the GBD database to assess the global HCV burden linked to obesity, acknowledging certain limitations. First, the GBD data is generated by integrating different types of data, including national survey data, epidemiological data, and processing missing data through various statistical methods. Nevertheless, many countries, particularly those with low and middle incomes, may have under-reporting or inadequate health monitoring. In such cases, the GBD data employs estimation methods to assess the burden of chronic diseases. This may lead to underestimation or overestimation of the disease burden, affecting the dynamic capture of disease and thus the representativeness of the study results. Additionally, while this study only examined individual etiologies, it did not account for the combined effects of multiple factors, such as obesity with type 2 diabetes or smoking and heavy alcohol use, nor did it consider the synergistic effects among various risk factors.

## 5 Conclusion

This study utilized the data from the GBD database in 2021 to evaluate the disease burden of HCV associated with HBMI. We found that this burden has been increasing over time from 1990 to 2021, with females experiencing a more severe impact than males. Additionally, the influence of obesity on this burden intensified with age. Globally, across various SDI regions and most GBD super-regions, the HCV burden related to HBMI exhibited a similar upward trend, moving further from frontier goals over time. Predictions for the next 15 years indicate that the impact of HBMI on HCV-infected patients will continue to rise. This underscores the critical role of weight management in hepatitis C prognosis. Consequently, targeted public health interventions are urgently needed, such as enhancing body fat management for individuals infected with the hepatitis C virus and promoting healthy eating habits and lifestyles. These measures are essential for alleviating the obesity burden on hepatitis C and are vital for achieving the global goal of eliminating hepatitis.

## Data Availability

The datasets presented in this study can be found in online repositories. The names of the repository/repositories and accession number(s) can be found in the article/Supplementary material.

## Footnotes

1 For detailed information, visit website https://www.who.int/news-room/fact-sheets/detail/hepatitis-c.

2 http://www.healthdata.org/research-analysis/gbd-data.

3 http://vizhub.healthdata.org/gbd-results.
