# Supplementary material for: Global burden of hepatitis C virus infection related to high body mass index and future forecast: an analysis based on the global burden of disease study 2021
Source: Front Public Health. 2025 Nov 20;13:1685807. doi: 10.3389/fpubh.2025.1685807 (PMC12675382; doi:10.3389/fpubh.2025.1685807)
Supplement: Supplementary file 3 [file Data_Sheet_2.PDF]

Supplement 1 The rank of risk factors that increase the burden of hepatitis C liver cancer

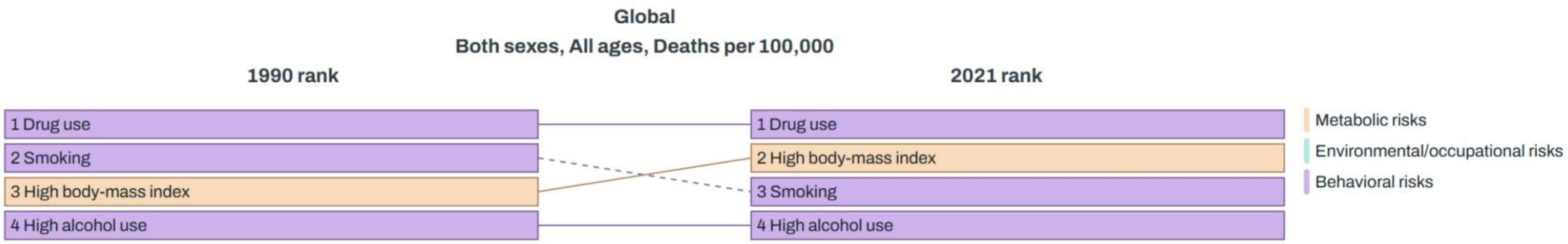

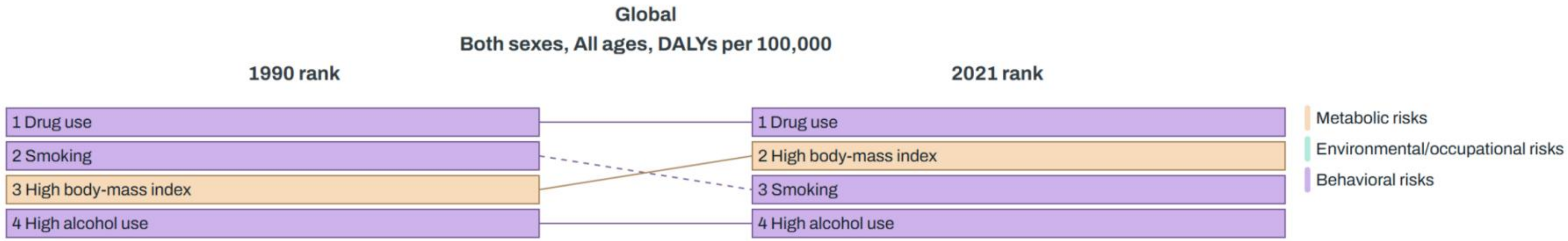

Supplement 2 The total burden of hepatitis C associated with HBMI in different genders and age groups in 2021

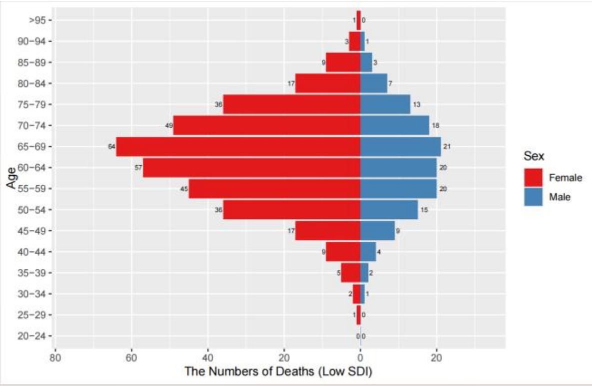

(a)

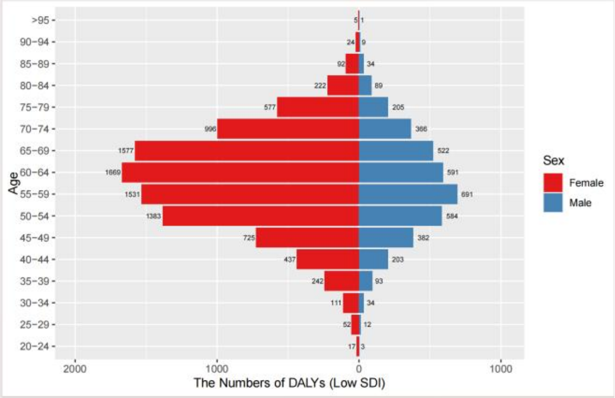

(b)

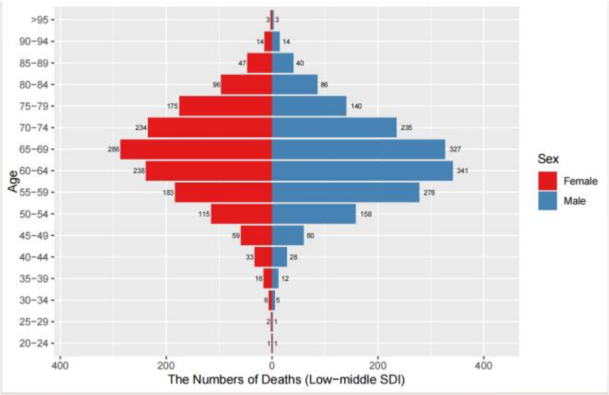

(c)

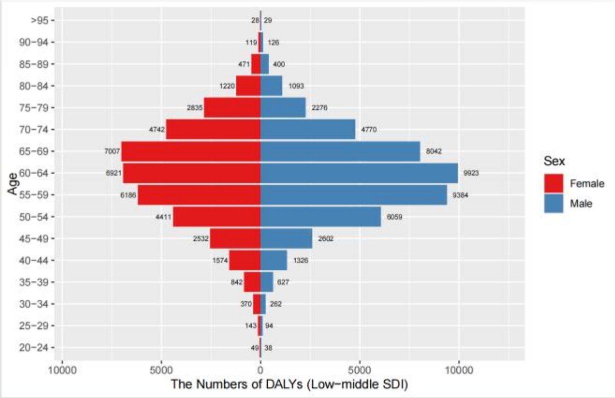

(d)

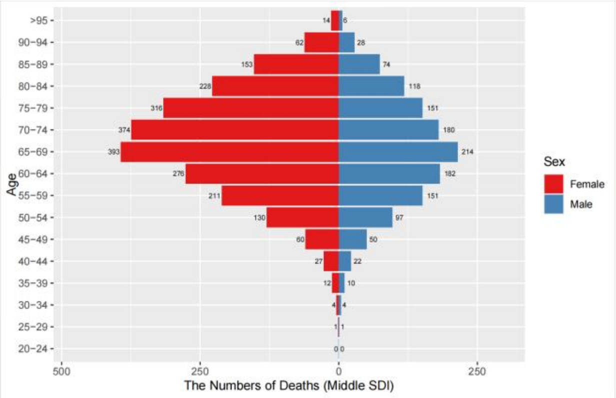

(e)

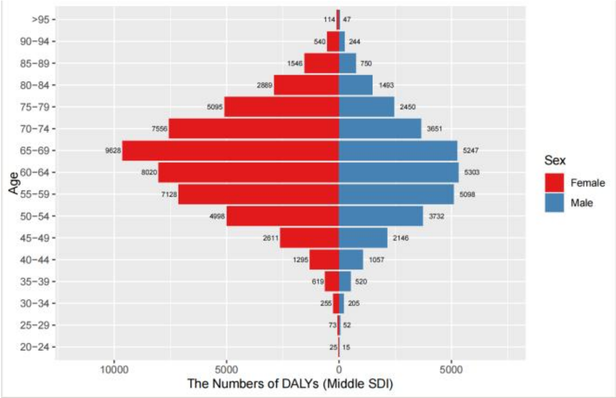

(f)

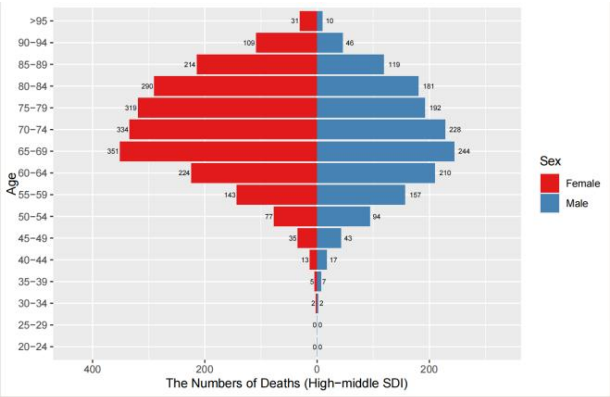

(g)

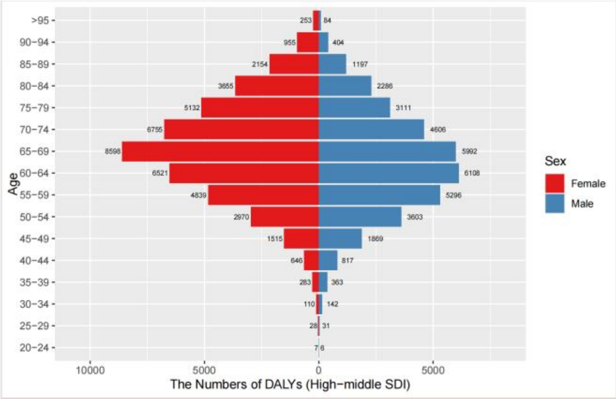

(h)

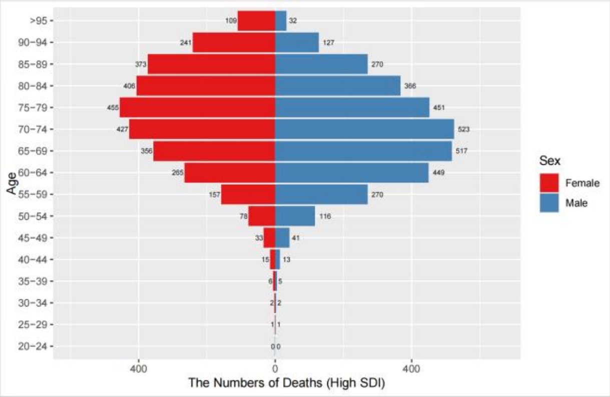

(i)

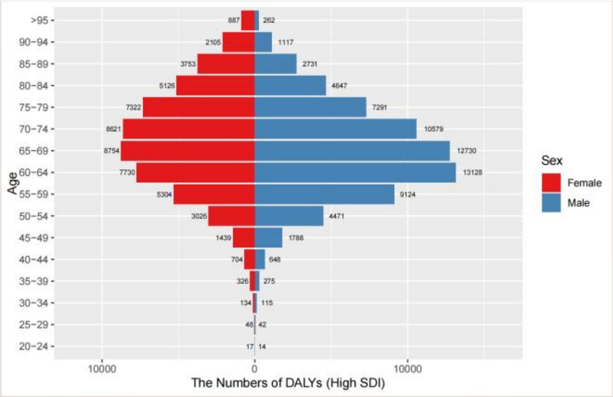

(j)

In Supplement 2, (a), (c), (e), (g), (i) represent the gender and age distributions of the numbers of Deaths in the Low SDI, Low - middle SDI, Middle SDI, High - middle SDI, and High SDI regions, respectively. Meanwhile, (b), (d), (f), (h), (j) represent the gender and age distributions of DALYs. Females are indicated in red, and males are indicated in blue.

Supplement 3 The ARIMA model of HCV disease burden related to HBMI in 2010

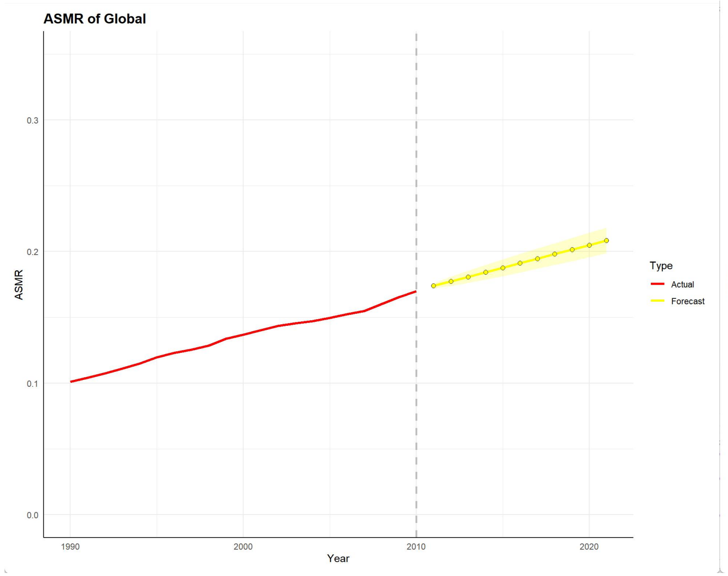

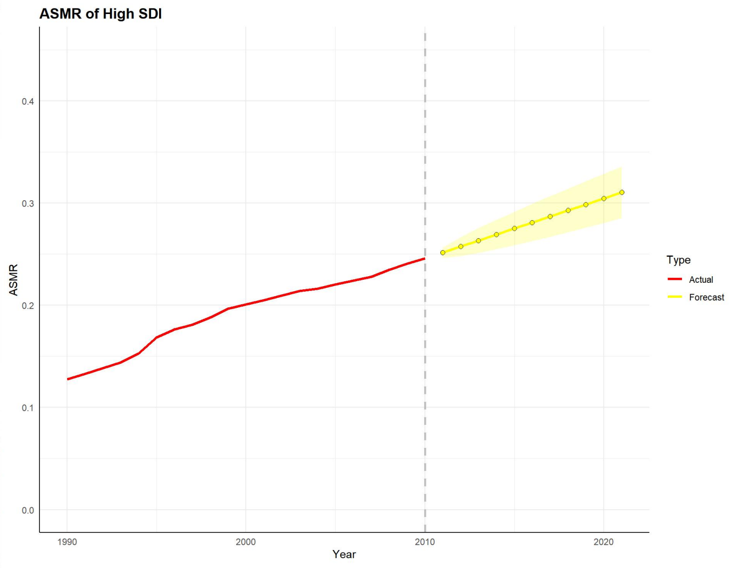

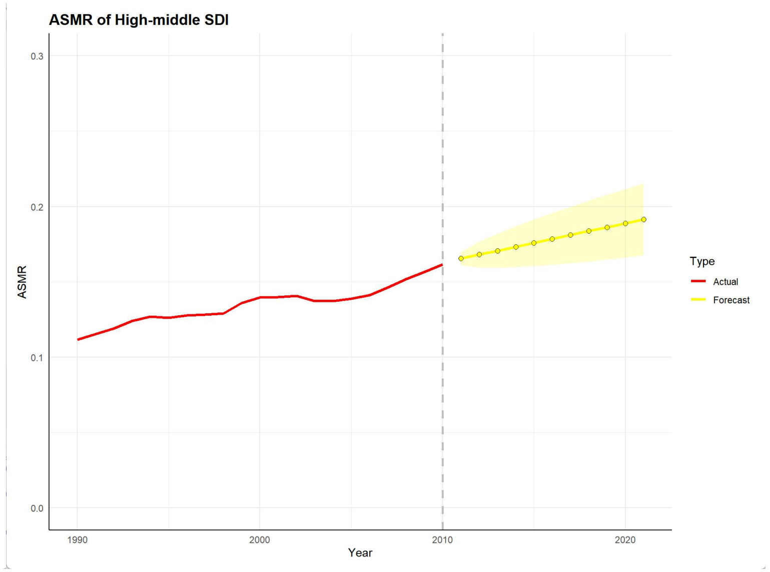

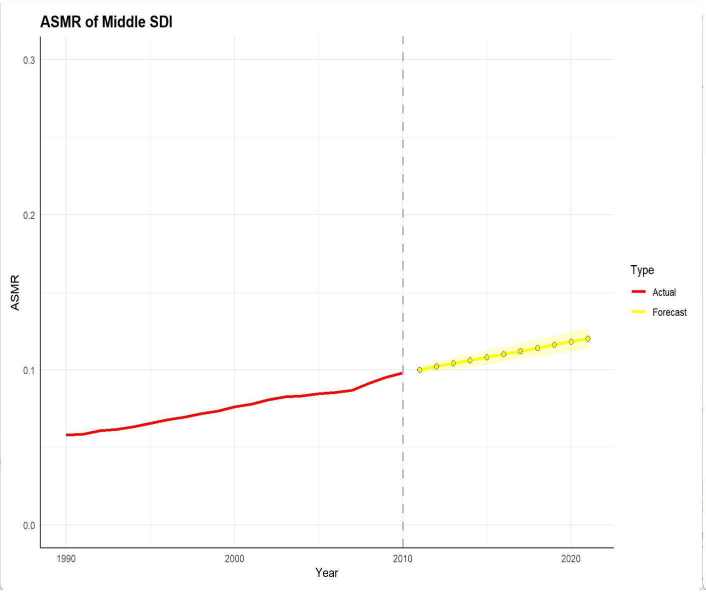

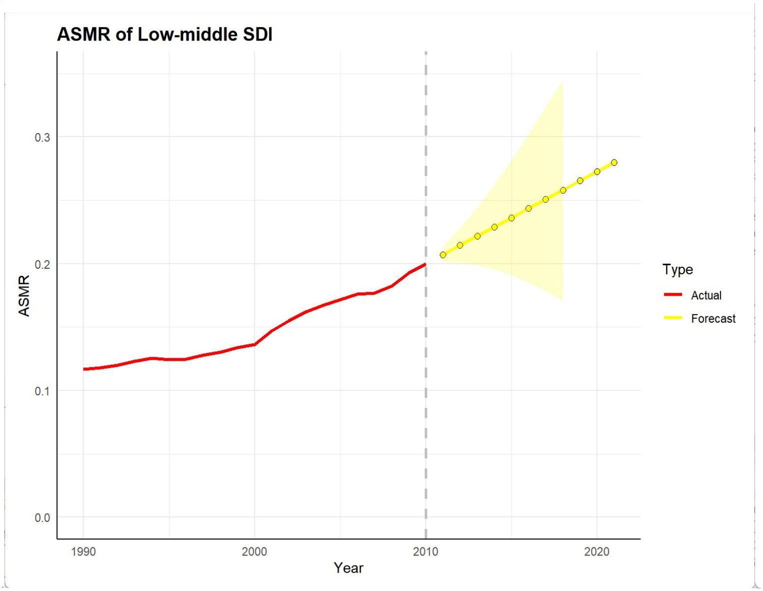

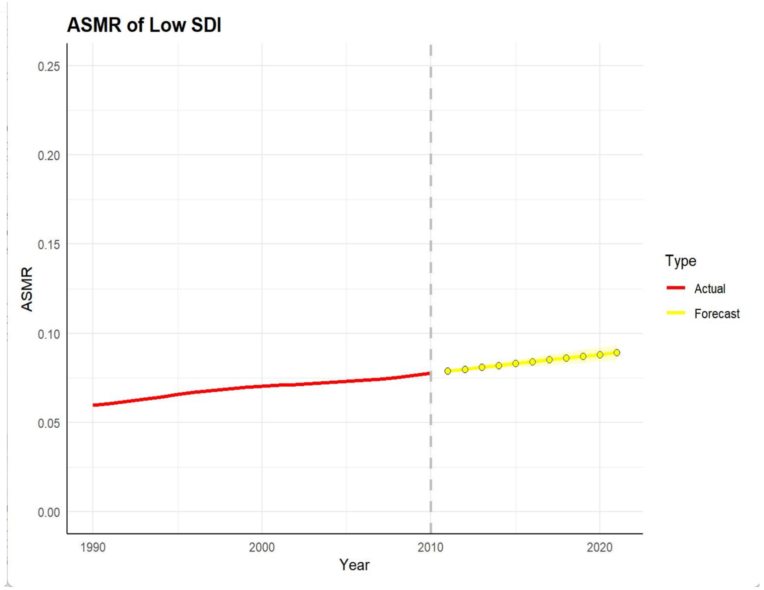

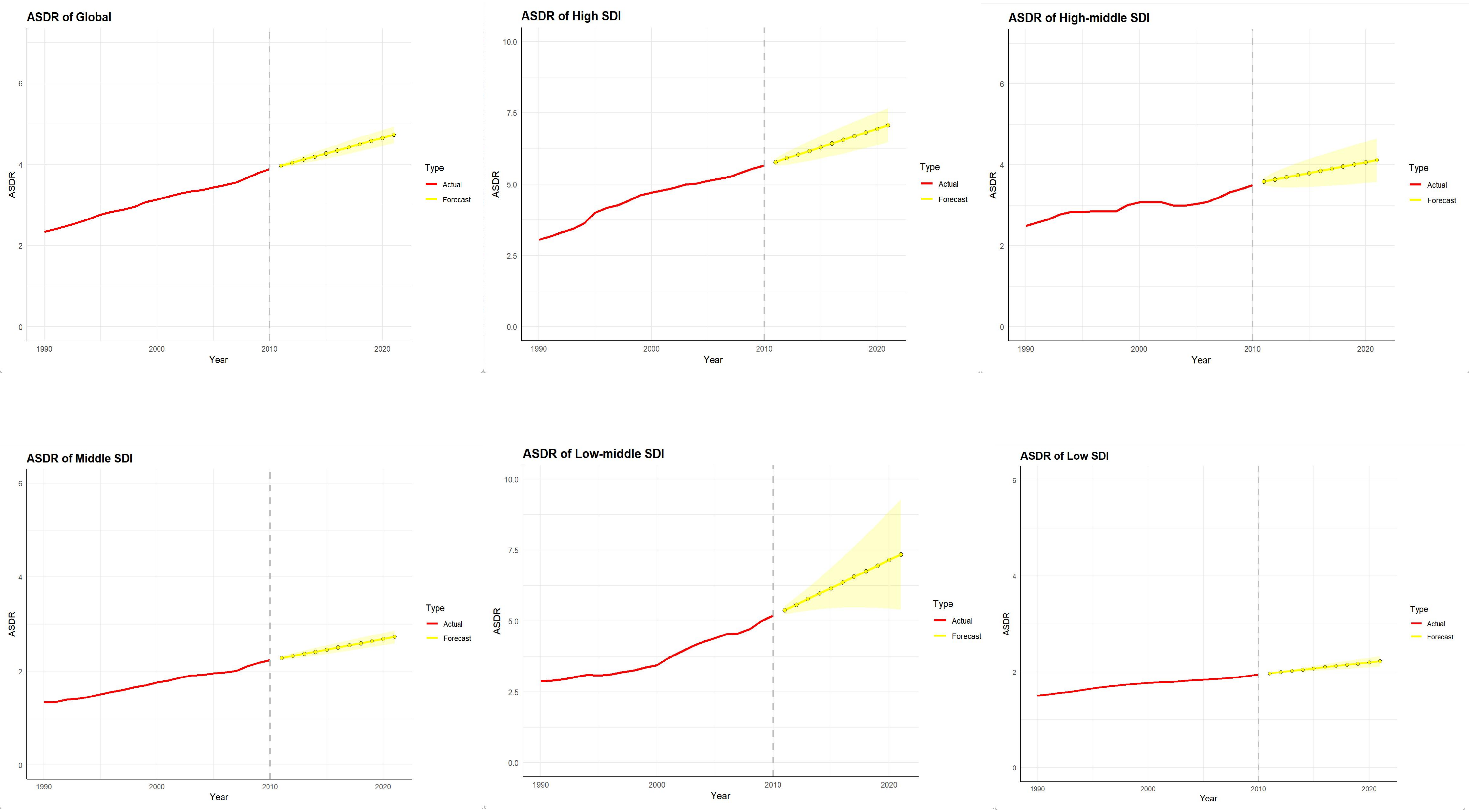
ASDR of Global
ASDR of High SDI
ASDR of High-middle SDI
ASDR of Middle SDI
ASDR of Low-middle SDI
ASDR of Low SDI
ASDR
Year
Type
Actual
Forecast
1990
2000
2010
2020
0
2
4
6
0.0
2.5
5.0
7.5
10.0

Supplement 4 A comparison between the predicted results and the actual results from 2011 to 2021

Location	Measure	2011	2012	2013	2014	2015	2016	2017	2018	2019	2020	2021	Z	P
Global	forecast	0.174 (0.172, 0.176)	0.177 (0.174, 0.181)	0.181 (0.176, 0.185)	0.184 (0.179, 0.190)	0.188 (0.181, 0.194)	0.191 (0.184, 0.198)	0.195 (0.187, 0.202)	0.198 (0.190, 0.206)	0.202 (0.193, 0.210)	0.205 (0.196, 0.214)	0.208 (0.199, 0.218)	-0.886	0.375
	actual	0.174	0.177	0.179	0.183	0.187	0.189	0.189	0.191	0.191	0.196	0.200		
High SDI	forecast	0.251 (0.246, 0.257)	0.257 (0.248, 0.267)	0.263 (0.251, 0.275)	0.269 (0.255, 0.284)	0.275 (0.259, 0.291)	0.281 (0.263, 0.299)	0.287 (0.267, 0.307)	0.293 (0.272, 0.314)	0.299 (0.276, 0.321)	0.305 (0.281, 0.328)	0.310 (0.285, 0.336)	-1.871	0.061
	actual	0.251	0.254	0.257	0.259	0.264	0.271	0.270	0.270	0.269	0.273	0.280		
High-middle SDI	forecast	0.165 (0.161, 0.170)	0.168 (0.159, 0.177)	0.171 (0.159, 0.182)	0.173 (0.160, 0.187)	0.176 (0.160, 0.191)	0.178 (0.161, 0.196)	0.181 (0.162, 0.200)	0.184 (0.163, 0.204)	0.186 (0.165, 0.208)	0.189 (0.166, 0.212)	0.191 (0.167, 0.215)	-1.083	0.279
	actual	0.165	0.169	0.171	0.176	0.175	0.171	0.174	0.177	0.178	0.183	0.186		
Middle SDI	forecast	0.100 (0.098, 0.102)	0.102 (0.099, 0.105)	0.104 (0.101, 0.108)	0.106 (0.102, 0.110)	0.108 (0.104, 0.113)	0.110 (0.105, 0.115)	0.112 (0.107, 0.117)	0.114 (0.108, 0.120)	0.116(0.110, 0.122)	0.118 (0.112, 0.125)	0.120 (0.113, 0.127)	-2.2	0.028
	actual	0.101	0.104	0.111	0.116	0.119	0.122	0.126	0.128	0.129	0.134	0.137		
Low-middle SDI	forecast	0.207(0.201, 0.213)	0.214 (0.201, 0.228)	0.222 (0.199, 0.245)	0.229 (0.196, 0.262)	0.236 (0.191, 0.282)	0.244 (0.185, 0.302)	0.251 (0.179, 0.323)	0.258 (0.171, 0.345)	0.265 (0.162, 0.368)	0.273 (0.153, 0.392)	0.280 (0.143, 0.417)	-2.659	0.008
	actual	0.207	0.210	0.205	0.210	0.222	0.223	0.217	0.218	0.218	0.222	0.227		
Low SDI	forecast	0.079 (0.078, 0.079)	0.080 (0.079, 0.081)	0.081 (0.080, 0.082)	0.082 (0.081, 0.084)	0.083 (0.081, 0.085)	0.084 (0.082, 0.087)	0.085 (0.082, 0.088)	0.086 (0.083, 0.089)	0.087 (0.084, 0.091)	0.088 (0.084, 0.092)	0.089 (0.085, 0.093)	-1.609	0.108
	actual	0.079	0.081	0.083	0.085	0.086	0.088	0.090	0.092	0.094	0.096	0.100		

Location	Measure	2011	2012	2013	2014	2015	2016	2017	2018	2019	2020	2021	Z	P
Global	forecast	3.963 (3.922, 4.003)	4.039 (3.964, 4.115)	4.116 (4.018, 4.214)	4.193 (4.076, 4.309)	4.269 (4.136, 4.402)	4.346 (4.199, 4.493)	4.422 (4.262, 4.583)	4.499 (4.327, 4.671)	4.576 (4.392, 4.759)	4.652 (4.458, 4.847)	4.729 (4.525, 4.933)	-1.083	0.279
	actual	3.965	4.036	4.072	4.157	4.257	4.293	4.278	4.299	4.299	4.375	4.461		
High SDI	forecast	5.774 (5.650, 5.899)	5.904 (5.680, 6.128)	6.034 (5.743, 6.325)	6.163 (5.818, 6.509)	6.293 (5.900, 6.686)	6.423 (5.988, 6.857)	6.552 (6.080, 7.025)	6.682 (6.174, 7.190)	6.812 (6.270, 7.353)	6.941 (6.369, 7.514)	7.071 (6.469, 7.673)	-2.397	0.017
	actual	5.755	5.823	5.881	5.908	6.003	6.141	6.106	6.065	6.016	6.073	6.215		
High-middle SDI	forecast	3.588 (3.499, 3.678)	3.641 (3.452, 3.830)	3.694 (3.441, 3.946)	3.746 (3.444, 4.049)	3.799 (3.454, 4.144)	3.852 (3.468, 4.235)	3.904 (3.486, 4.323)	3.957 (3.507, 4.407)	4.010 (3.530, 4.490)	4.063 (3.554, 4.571)	4.115 (3.580, 4.650)	-0.755	0.45
	actual	3.562	3.647	3.684	3.803	3.807	3.740	3.776	3.846	3.864	3.970	4.019		
Middle SDI	forecast	2.279 (2.237, 2.320)	2.324 (2.265, 2.382)	2.369 (2.297, 2.440)	2.414 (2.332, 2.496)	2.459 (2.367, 2.551)	2.504 (2.403, 2.604)	2.549 (2.440, 2.657)	2.594 (2.478, 2.710)	2.639 (2.516, 2.762)	2.684 (2.554, 2.814)	2.729 (2.593, 2.865)	-2.134	0.033
	actual	2.296	2.377	2.502	2.611	2.702	2.776	2.834	2.884	2.920	3.003	3.058		
Low-middle SDI	forecast	5.380 (5.236, 5.524)	5.576 (5.312, 5.840)	5.773 (5.374, 6.172)	5.969 (5.421, 6.518)	6.166 (5.454, 6.878)	6.362 (5.474, 7.251)	6.559 (5.481, 7.636)	6.755 (5.478, 8.032)	6.952 (5.463, 8.440)	7.148 (5.438, 8.858)	7.345 (5.403, 9.286)	-3.119	0.002
	actual	5.349	5.422	5.296	5.407	5.712	5.697	5.528	5.526	5.506	5.587	5.713		
Low SDI	forecast	1.968 (1.958, 1.978)	1.995 (1.975, 2.014)	2.021 (1.991, 2.051)	2.047 (2.006, 2.087)	2.072 (2.020, 2.123)	2.096 (2.035, 2.158)	2.121 (2.049, 2.193)	2.145 (2.064, 2.226)	2.170 (2.079, 2.260)	2.194 (2.095, 2.292)	2.217 (2.110, 2.325)	-1.609	0.108
	actual	1.973	2.014	2.068	2.119	2.153	2.188	2.232	2.280	2.328	2.393	2.474		

Supplement 3 is to use the data of HCV disease burden related to HBMI in 2010 to predict ASMR and ASDR in the next 11 years. The red line represents the actual trends of ASMR and ASDR from 1990 to 2010, and the yellow dashed line and shaded area represent the predicted trends and their 95% CIs. Supplement 4 is a comparison between the predicted results and the actual results from 2011 to 2021.
